# Supplementary figures and images for: Optogenetic and chemogenetic approaches reveal differences in neuronal circuits that mediate initiation and maintenance of social interaction
Source: PLoS Biol. 2023 Nov 29;21(11):e3002343. doi: 10.1371/journal.pbio.3002343 (PMC10686636; doi:10.1371/journal.pbio.3002343)

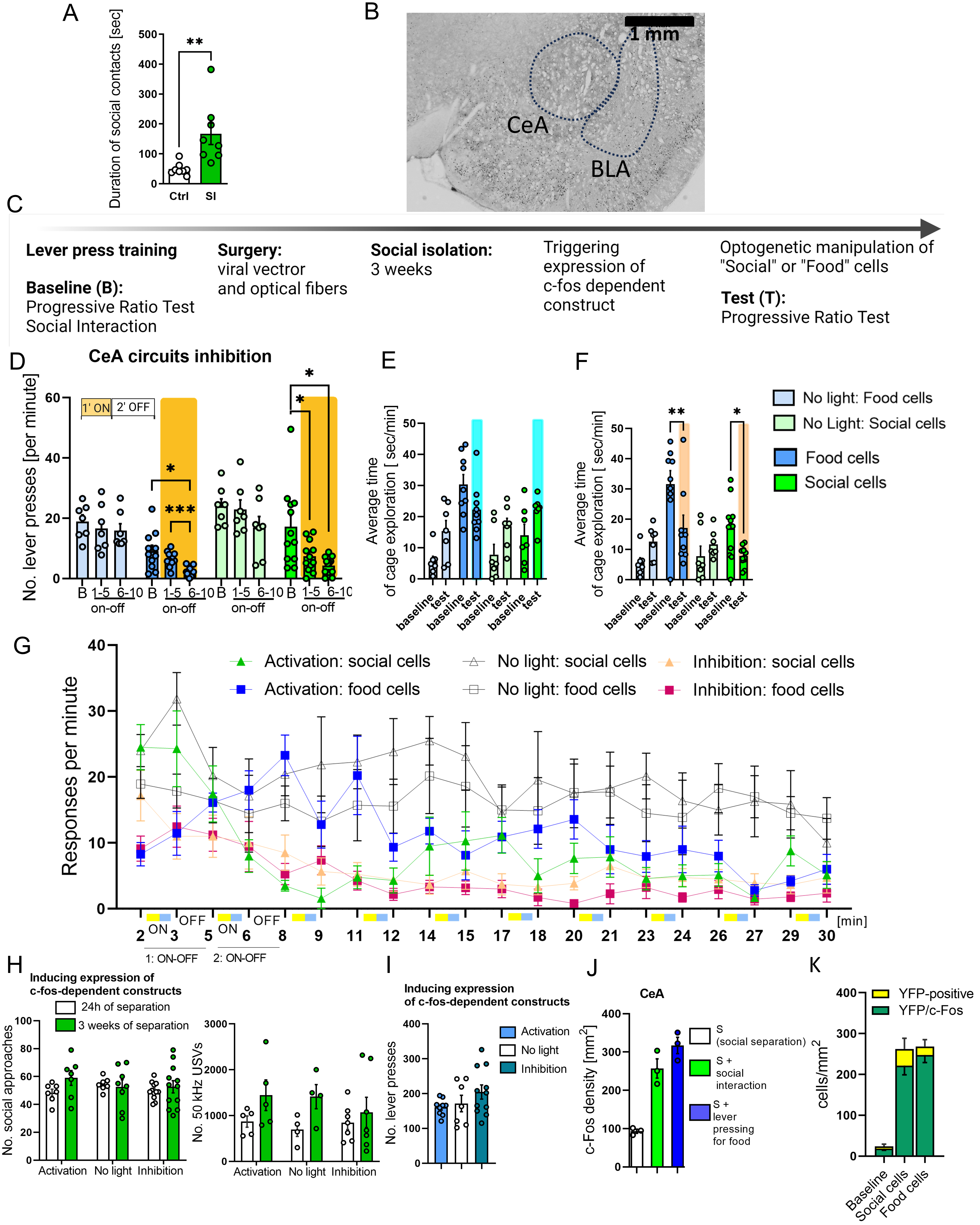

Supplement: S1 Fig — (A) Duration of social contacts during the 10-minute social interaction (social contacts: SI, n = 8; Ctrl, n = 8; social contact: unpaired t test: t(14) = 3.233, p = 0.006). Cagemate rats were separated for 3 weeks and then subjected to the social interaction (SI group). The control group had the social interaction after a brief, 10-minute separation (Ctrl) (B) Representative image of c-Fos expression in the CeA in the SI group (lower magnification of the image shown in Fig 1C). (C) The experimental schematic. (D) Inhibition of both the food and social cells decreased lever pressing compared to baseline; in controls, there was no difference between the baseline and following phases; two-way ANOVA (time effect: F(1.460,48,17) = 12.40, p = 0.0002), followed by Holm–Sidak post hoc tests. The average number of lever presses per minute during the baseline period (2 minutes) and ON–OFF (3 minutes) laser periods are shown. Ctrl: Social cells: n = 7, Ctrl: Food cells: n = 7, Social cells: Inhibition: n = 12, Food cells: Inhibition: n = 11. (E) Time spent on exploration when the CeA cells were activated (baseline: 2 minutes, test: 6 minutes) (F) and inhibited (baseline: 2 minutes, test: 28 minutes). Inhibition: two-way ANOVA (time × group effect: F(3,31) = 6.437, p = 0.0016), followed by Holm–Sidak post hoc tests. No light: Social cells n = 7, No light: Food cells: n = 7, Social cells: Activation/Inhibition: n = 7/12, Food cells: Activation/Inhibition: n = 9/9. (G) The overall rate of lever presses per minute throughout the entire testing session when the CeA cells were activated or inhibited. (H) The number of social contacts and 50 kHz ultrasonic vocalizations did not differ between the groups during social interaction inducing expression of c-fos–dependent constructs. Social approaches: No light: n = 7, Activation/Inhibition: n = 7/12, USVs: No light: n = 4 pairs, Activation/Inhibition: n = 5/7 pairs. (I) The number of lever presses did not differ between the groups durin [file pbio.3002343.s001.tif]

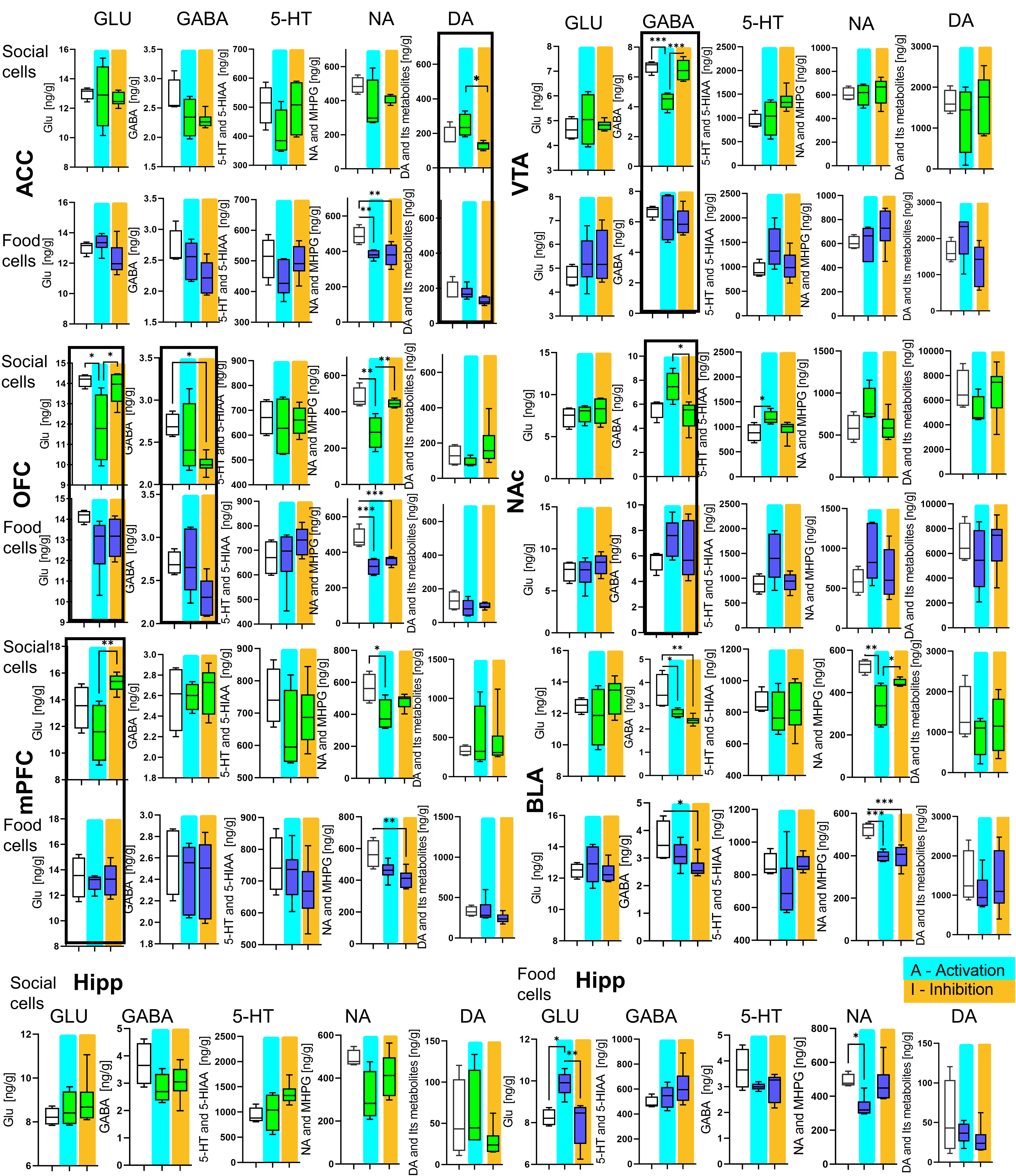

Supplement: S2 Fig — Activation (blue background) or Inhibition (yellow background) of the CeA social cells. Ctrl: n = 4, Social cells: Activation/Inhibition: n = 4/6, Food cells: Activation/Inhibition: n = 6/6. Black frames indicate the difference in neurotransmitter levels and their metabolites after manipulating the CeA social but not food cells. ACC; social cells-DA; Kruskal–Wallis test (p = 0.0039) followed by Dunn post hoc tests. Food cells-NA; one-way ANOVA (group effect: F(2,13) = 8.344, p = 0.0047) followed by Holm–Sidak post hoc tests. OFC: social cells-Glu; one-way ANOVA (group effect: F(2,11) = 6.258, p = 0.0153) followed by Holm–Sidak post hoc tests. Social cells-GABA; one-way ANOVA (group effect: F(2,11) = 4.583, p = 0.0357) followed by Holm–Sidak post hoc tests. Social cells-NA; one-way ANOVA (group effect: F(2,11) = 13.59, p = 0.0011) followed by Holm–Sidak post hoc tests. Food cells-NA; one-way ANOVA (group effect: F(2,13) = 18.88, p = 0.0001) followed by Holm–Sidak post hoc tests. mPFC: social cells-Glu; one-way ANOVA (group effect: F(2,11) = 7.530, p = 0.0087) followed by Holm–Sidak post hoc tests. Social cells-NA; one-way ANOVA (group effect: F(2,11) = 5.326, p = 0.0241) followed by Holm–Sidak post hoc tests. Food cells-NA; one-way ANOVA (group effect: F(2,13) = 6.979, p = 0.0087) followed by Holm–Sidak post hoc tests. VTA: Social cells-GABA; one-way ANOVA (group effect: F(2,11) = 19.91, p = 0.0002) followed by Holm–Sidak post hoc tests. Hipp: Food cells-Glu; one-way ANOVA (group effect: F(2,13) = 10.42, p = 0.002) followed by Holm–Sidak post hoc tests. Food cells-NA; Kruskal–Wallis test (p = 0.0023) followed by Dunn post hoc tests. BLA: Social cells-GABA; one-way ANOVA (group effect: F(2,11) = 10.75, p = 0.0026) followed by Holm–Sidak post hoc tests. Social cells-NA; one-way ANOVA (group effect: F(2,11) = 10.45, p = 0.0029) followed by Holm–Sidak post hoc tests. Food cells-GABA; Kruskal–Wallis test (p = 0.0352) followed by Dunn post hoc tests. Food cells-NA; one-way [file pbio.3002343.s002.tif]

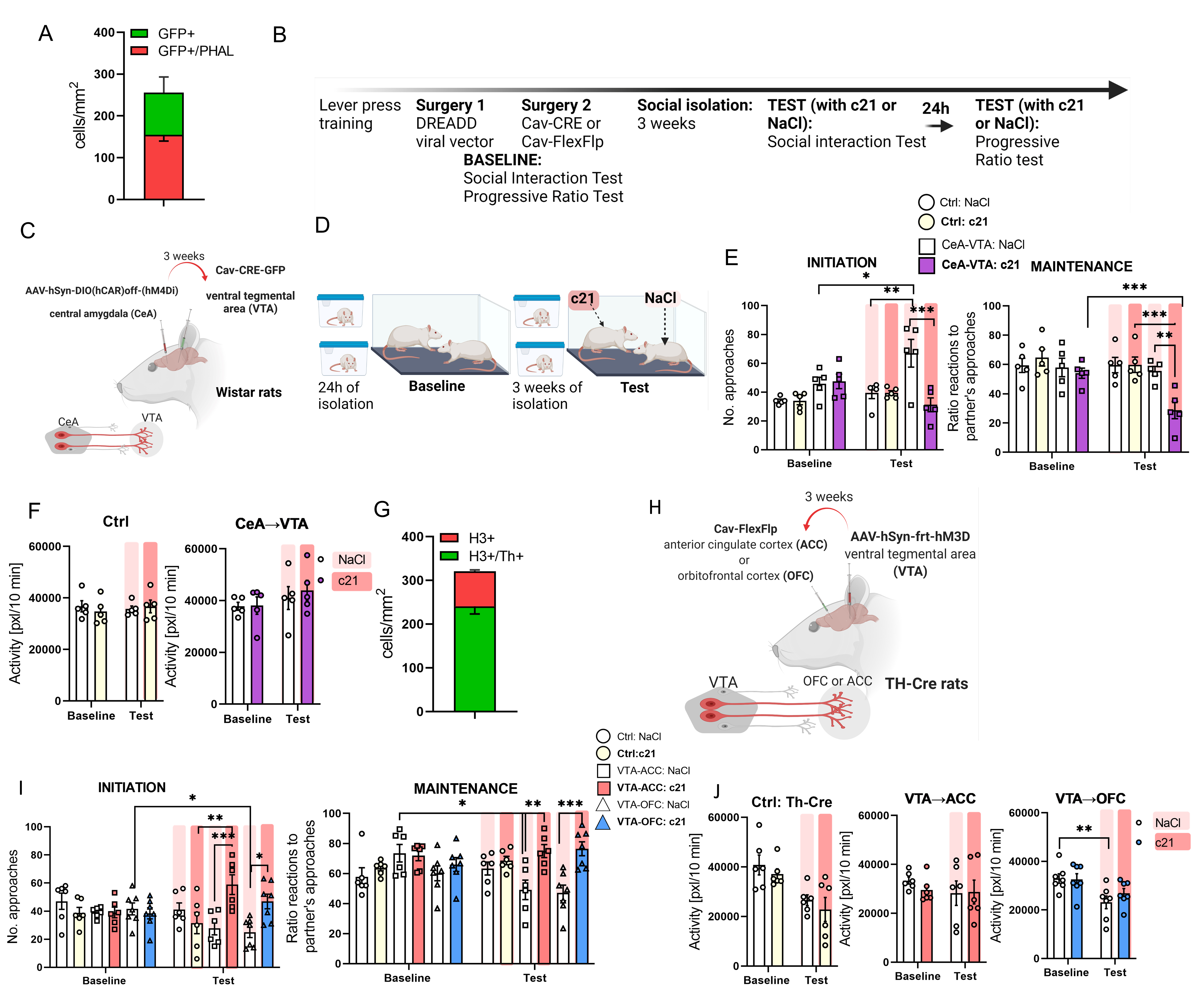

Supplement: S3 Fig — (A) Quantification of CeA projections to VTA neurons activated by social interaction (n = 3). (B) The timeline of the experiments. (C) Chemogenetic inhibition schematic. (D) Schematic of the experiment. The baseline of social interaction was measured after 24 hours of social separation. The test was performed after 3 weeks of social isolation. Before the test, one rat from the pair was injected with c21, and his partner was injected with NaCl. (E) Comparison to the baseline: Inhibition of the CeA-VTA projection disrupts the maintenance of social interaction. Initiation: two-way ANOVA (test × group effect: F(5,28) = 8.376, p < 0.0001) followed by Holm–Sidak post hoc tests. Maintenance: two-way ANOVA (group effect: F(3,16) = 5.90, p = 0.0065) followed by Holm–Sidak post hoc tests. (F) Activity after CeA-VTA inhibition. Ctrl: NaCl/c21 n = 5/5, CeA-VTA: NaCl/c21 n = 5/5. (G) Quantification of H3 expression in VTA Th-positive cells (n = 2). (H) Chemogenetic activation schematic. (I) Comparison to the baseline: The activation of the VTA-OFC dopaminergic projection decreases the initiation of social interaction by the partner rat, whereas the activation of the VTA-ACC pathway affects the maintenance of social contact by the partner rat. Initiation: two-way ANOVA (test × group effect: F(3,16) = 5.865, p = 0.0067) followed by Holm–Sidak post hoc tests. Maintenance: two-way ANOVA (test × group effect: F(5,32) = 3.469, p = 0.0129) followed by Holm–Sidak post hoc tests. (J) Activity after VTA-ACC and VTA-ACC activation. VTA-OFC two-way ANOVA (test: F(1,12) = 15.35, p = 0.0020), followed by Holm–Sidak post hoc tests. Ctrl: NaCl/c21 n = 6/6, VTA-ACC: NaCl/c21 n = 6/6, VTA-OFC: NaCl/c21 n = 7/7. Pink background: rats injected with c21 before test, light pink background: rats injected with NaCl before test. All the data are shown as the mean ± SEM; dots represent individual data points, * p < 0.05, ** p < 0.01, *** p < 0.001. The data underlying this figure can be found in https:// [file pbio.3002343.s003.tif]

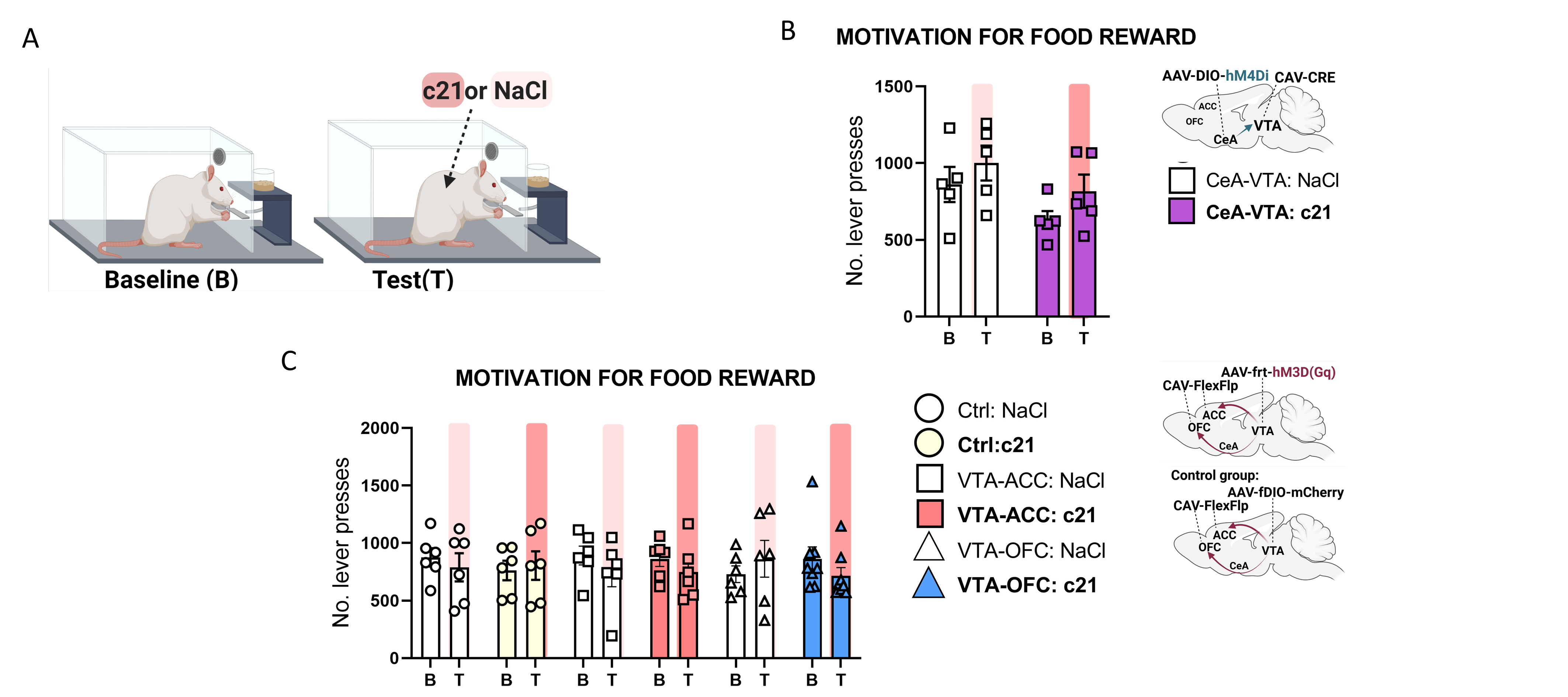

Supplement: S4 Fig — (A) The schematic of the experiment. Rats were tested in the Progressive Ratio test to assess their motivation for obtaining food reward (sucrose pellet). The Baseline (B) measurement was taken before social separation, and the test was performed 1 day after the social interaction test. Before the test animals were injected with either c21 (pink background) or NaCl (light pink background, ctrl). (B) Inhibition of the CeA-VTA has no effect on the number of lever presses for food. Comparison between test (with c21 or NaCl) and baseline. Ctrl: NaCl/c21 n = 5/5, CeA-VTA: NaCl/c21 n = 5/5. (C) Activation of either the VTA-ACC or VTA-OFC has no effect on the number of lever presses for food. Comparison between test (with c21 or Nacl) and baseline; Ctrl: NaCl/c21 n = 6/6, VTA-ACC: NaCl/c21 n = 6/6, VTA-OFC: NaCl/c21 n = 6/8. All the data are shown as the mean ± SEM, and symbols represent individual data points, ** p < 0.01. The data underlying this figure can be found in https://data.mendeley.com/datasets/h49vtpjm8f/3. (TIF) [file pbio.3002343.s004.tif]

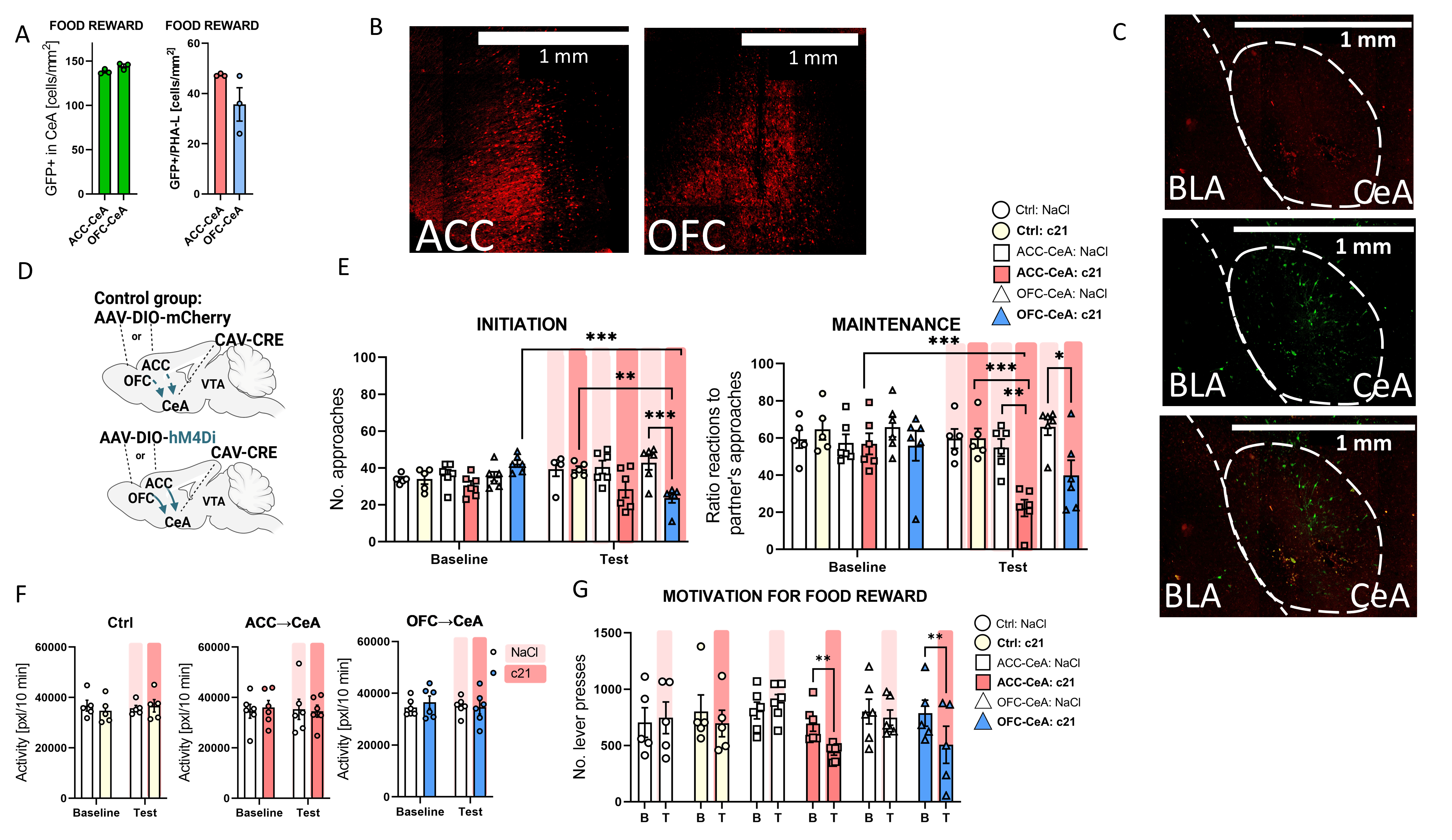

Supplement: S5 Fig — (A) Quantification of CeA neurons activated by lever pressing for food (left) and neurons that receive projections from ACC or OFC (right); ACC-CeA: n = 3, OFC-CeA: n = 3. (B) The representative images of the AAV-hSyn-DIO-{hCAR}off-{hM4Di-mCherry}on-W3SL expression in the ACC and OFC (lower magnification of the images shown in Fig 4G). (C) The example of image with ACC inputs in the CeA with expression of AAV-DIO-mCherry ACC (top) and expression of Cav-Cre-GFP in the the CeA (middle) and the merged image of all the former (down). (D) Chemogenetic inhibition schematic. (E) Comparison to the baseline: (Left) Chemoinhibition of the OFC-CeA projection decreases the number of social approaches to partner. (Right) Inhibition of the ACC-CeA pathway decreases the maintenance of social interaction. Initiation: two-way ANOVA (test × group effect: F(5,28) = 8.376, p < 0.0001) followed by Holm–Sidak post hoc tests. Maintenance: two-way ANOVA (test × group effect: F(5,28) = 3.374, p = 0.0165) followed by Holm–Sidak post hoc tests. (F) Locomotor activity after ACC-CeA and OFC-CeA inhibition. Ctrl: NaCl/c21 n = 5/5, ACC-CeA: NaCl/c21 n = 6/6, OFC-CeA: NaCl/c21 n = 6/6. (G) Inhibition of the ACC-CeA and OFC-CeA projections decreases the number of lever presses for food. Comparison between test (with c21 or Nacl) and baseline; two-way ANOVA (test × group effect: F(5,27) = 4.319, p = 0.0051), followed by Holm–Sidak post hoc tests; Ctrl: NaCl/c21 n = 5/5, ACC-CeA: NaCl/c21 n = 6/6, OFC-CeA: NaCl/c21 n = 6/5. Pink background: rats injected with c21 before test, light pink background: rats injected with NaCl before test. All the data are shown as the mean ± SEM; dots represent individual data points, ** p < 0.01, *** p < 0.001. The data underlying this figure can be found in https://data.mendeley.com/datasets/h49vtpjm8f/3. (TIF) [file pbio.3002343.s005.tif]
