# Supplementary material for: Optogenetic and chemogenetic approaches reveal differences in neuronal circuits that mediate initiation and maintenance of social interaction
Source: PLoS Biol. 2023 Nov 29;21(11):e3002343. doi: 10.1371/journal.pbio.3002343 (PMC10686636; doi:10.1371/journal.pbio.3002343)
Supplement: S1 Table — (DOCX) [file pbio.3002343.s006.docx]

**S1 Table**

| REAGENT OR RESOURCE | SOURCE | IDENTIFIER |
| --- | --- | --- |
| ANTIBODIES | | |
| Primary: anti-c-Fos rabbit antibody 1:1000 | Millipore | Cat# ABE-457 |
| Primary: Anti-Green Fluorescent Protein Antibody 1:500 | Millipore | Cat# MAB3580 |
| Primary: Anti-Th mouse antibody 1:500 | Millipore | Cat# MAB5280 |
| Primary: anti-mCherry chicken Antibody (1;500) | Novus Biologicals | Cat# NBP2-25158 |
| Primary: Anti-mCherry rabbit antibody (1:500) | Abcam | Cat# ab167453 |
| Primary: anti-Anti-GAD67 rabbit antibody (1:500) | Abcam | Cat# ab213508 |
| Secondary: antibody conjugated to Alexa Fluor 488 made in mouse 1:500 | Invitrogen | Cat# A32723 |
| Secondary: antibody conjugated to Alexa 594 made in rabbit 1:500 | Invitrogen | Cat# A32740 |
| Secondary: antibody anti-Chicken IgY  conjugated to Alexa Fluor 555 made in goat 1:500 | ThermoFisher Scientific | Cat# A-21437 |
| Secondary: antibody anti-rabbit IgY  conjugated to Alexa Fluor 647 made in goat 1:500 | ThermoFisher Scientific | Cat# A-21244 |
| **CHEMICALS, PEPTIDES AND RECOMBINANT PROTEINS** | | |
| Phusion High-Fidelity DNA Polymerase | ThermoFisher Scientific | Cat# F530S |
| One Shot Stbl3 | ThermoFisher Scientific | Cat# C737303 |
| Normal Goat Serum | Abcam | Cat# ab7481 |
| TRIS | Millipore | Cat# 1083821000 |
| Blocker™ BSA (10X) in PBS | ThermoFisher Scientific | Cat# 37525 |
| EndoFree Maxi Prep | Qiagen | Cat# 12362 |
| KCl (50 mM) | Sigma | Cat# P9541 |
| PHA-L Alexa Fluor 647 conjugate | Invitrogen; Molecular Probes | Cat# L-32457 |
| PBS Tablets (pH 7.4) | ThermoFisher Scientific | Cat# 18912014 |
| paraformaldehyde | POCh | Cat# 715400427 |
| Triton X-100 | Polysciences | Cat# 04605 |
| Blocker™ BSA (10X) in PBS | ThermoFisher Scientific | Cat# 37525 |
| Entellan mounting medium | Merck | Cat# 107961 |
| DAPI Fluoromount-G | SouthernBiotech | Cat# 0100-20 |
| C21 - DREADD agonist 21 (Compound 21) dihydrochloride (water soluble) | Hellobio | Cat# HB6124 |
| 45 mg sucrose pallet | Bio-Serv | Cat# F06233 |
| 3,4-dihydroxyphenylacetic acid | Merck | Cat# 11569-25MG |
| homovanillic acid | Merck | Cat# 69673-25MG |
| 3-methoxytyramine | Merck | Cat# 75024-25MG |
| **BACTERIAL AND VIRUS STRAINS** | | |
| pAAV-hSyn-DIO {hCAR}off-{hM4Di-mCherry}on-W3SL | AddGene | Cat# 111397 |
| AAV-hSyn-DIO-mCherry | AddGene | Cat# 50459-AAV9 |
| Cav2-CreGFP | Plateforme de Vectorologie de Montpellier |  |
| AAV-c-fos-ChR2-EYFP | Nencki Institute of Experimental Biology, Laboratory of Animal Models | [1] |
| AAV-c-fos-NpHR-EYFP | Nencki Institute of Experimental Biology, Laboratory of Animal Models | [1] |
| Cav2- FlxFlp | Plateforme de Vectorologie de Montpellier |  |
| pAAV-EF1a-DIO-hM3D(Gq)-mCherry | Division of Neuroscience, Department of Translational Neuroscience, Brain Center Rudolf Magnus, University Medical Center Utrecht, Utrecht, Netherlands | [2] |
| AAV-Ef1a-fDIO mCherry | AddGene | Cat# 114471-AAV5 |
| **EXPERIMENTAL MODELS: ORGANISM/STRAINS** | | |
| c-fos-PSD95Venus-Arc Rats | Nencki Institute Animal House or Faculty of Biology Animal House (University of Warsaw), Poland | Venus |
| Wistar Rats | Center of Experimental Medicine in Bialystok, Poland | Wistar |
| Tyrosine hydroxylase (TH) IRES-Cre rats^+/-^ transgenic rats | Institute of Pharmacology PAS breeding facility (Krakow, Poland) | Sprague Dawley |
| **SOFTWARE:** | | |
| Fiji/ImageJ 1.52 – image analysis | National Institutes of Health, USA | https://imagej.net/Fiji |
| Image-Pro Plus 7.0.1.658 – image capture | Media Cybernetics | https://www.mediacy.com/imageproplus |
| Med-PC IV – behavioral 2data collection | Med-Associates | https://www.med-associates.com |
| GraphPad Prism version 9 –statistical analysis | GraphPad Software, Inc., San Diego, CA | https://www.graphpad.com/ |
| BehaView Software – behavior analysis | Paweł Boguszewski | http://www.pmbogusz.net/?a=behaview |
| RAVEN PRO | Cornell Lab of Ornithology | https://ravensoundsoftware.com/software/ |
| Bonsai open-source software | Bonsai | https://bonsai-rx.org/  [3] |

Reference

1. Andraka K, Kondrakiewicz K, Rojek-Sito K, Ziegart-Sadowska K, Meyza K, Nikolaev T, et al. Distinct Circuits in Rat Central Amygdala for Defensive Behaviors Evoked by Socially Signaled Imminent Versus Remote Danger. Current Biology. 2021; S0960982221004206. doi:10.1016/j.cub.2021.03.047
2. Kakava-Georgiadou N, Zwartkruis MM, Bullich-Vilarrubias C, Luijendijk MCM, Garner KM, van der Plasse G, et al. An Intersectional Approach to Target Neural Circuits With Cell- and Projection-Type Specificity: Validation in the Mesolimbic Dopamine System. Front Mol Neurosci. 2019;12: 49. doi:10.3389/fnmol.2019.00049
3. Lopes G, Bonacchi N, FrazÃ£o J, Neto JP, Atallah BV, Soares S, et al. Bonsai: an event-based framework for processing and controlling data streams. Front Neuroinform. 2015;9. doi:10.3389/fninf.2015.00007
