# Supplementary material for: Optogenetic and chemogenetic approaches reveal differences in neuronal circuits that mediate initiation and maintenance of social interaction
Source: PLoS Biol. 2023 Nov 29;21(11):e3002343. doi: 10.1371/journal.pbio.3002343 (PMC10686636; doi:10.1371/journal.pbio.3002343)
Supplement: S2 Table — Avoidance behavior during the inhibition of the ACC-CeA projection in response to the partner’s attempts to initiate social contact (the focal rat marked in red, the partner rat marked in green). Pushing-back behavior during the inhibition of the ACC-CeA projection in response to the partner’s attempts to initiate social contact (the focal rat marked in red, the partner rat marked in green). (DOCX) [file pbio.3002343.s007.docx]

**S2 Table**

| **Fig** | **Title** | **group** | **test** | **p** | **t,df** |  |  |
| --- | --- | --- | --- | --- | --- | --- | --- |
| F1 B | No. social contact | Ctrl vs. SI | Unpaired t test | p<0,0001 | t=5,588, df=14 |  |  |
| F1 B | No. USVs | Ctrl vs. SI | Unpaired t test | p=0,0068 | t=3,076, df=17 |  |  |
| F1 D | c-Fos | Ctrl vs. SI | Unpaired t test | p<0,0001 | t=11,63, df=14 |  |  |
| F1 G | % laser | on vs. off | Paired t test | p<0,0001 | t=17,00, df=5 |  |  |
| F1 H | % laser Ctrl | on vs. Off | Paired t test | p=0,654 | t=0,4714, df=6 |  |  |
| F1 K | **ChR2** | **Two-way RM ANOVA** | **DF** | **MS** | **F (DFn, DFd)** | **P value** |  |
|  |  | time x group | 2059 | 6 | 343,2 | F (6, 52) = 8,052 | P<0,0001 |
|  |  | time | 350,2 | 2 | 175,1 | F (1,747, 45,43) = 4,108 | P=0,0275 |
|  |  | group | 927,9 | 3 | 309,3 | F (3, 26) = 3,487 | P=0,0299 |
|  |  | Subject | 2306 | 26 | 88,7 | F (26, 52) = 2,081 | P=0,0123 |
|  | **Holm-Šídák's multiple comparisons test** | **Predicted (LS) mean diff,** | **Below threshold?** | **Summary** | **Adjusted P Value** |  |  |
|  | Light: social cells |  | | | |  | |
|  | Baseline vs. on-off:1 | 3,643 | No | ns | 0,4317 |  |  |
|  | Baseline vs. on-off:2 | 18,71 | Yes | * | 0,0172 |  |  |
|  | on-off:1 vs. on-off:2 | 15,07 | Yes | * | 0,0467 |  |  |
|  | Light: non-social cells |  |  |  |  |  |  |
|  | Baseline vs. on-off:1 | -5,556 | No | ns | 0,0958 |  |  |
|  | Baseline vs. on-off:2 | -12,08 | Yes | * | 0,0129 |  |  |
|  | on-off:1 vs. on-off:2 | -6,528 | No | ns | 0,0958 |  |  |
|  | Ctrl: Food cells |  | | | |  |  |
|  | Baseline vs. on-off:1 | 1,786 | No | ns | 0,731 |  |  |
|  | Baseline vs. on-off:2 | 3,714 | No | ns | 0,731 |  |  |
|  | on-off:1 vs. on-off:2 | 1,929 | No | ns | 0,731 |  |  |
|  | Ctrl: Social cells |  | | | |  |  |
|  | Baseline vs. on-off:1 | -2,071 | No | ns | 0,1415 |  |  |
|  | Baseline vs. on-off:2 | 5,286 | No | ns | 0,1415 |  |  |
|  | on-off:1 vs. on-off:2 | 7,357 | No | ns | 0,0781 |  |  |
| F2 D | **Initiation** | **ANOVA table** | **SS** | **DF** | **MS** | **F (DFn, DFd)** | **P value** |
|  |  | Column Factor | 3675 | 3 | 1225 | F (3, 16) = 7,364 | P=0,0026 |
|  | **Holm-Šídák's multiple comparisons test** | **Predicted (LS) mean diff,** | **Below threshold?** | **Summary** | **Adjusted P Value** |  |  |
|  | Ctrl: nacl vs. Ctrl: c21 | 0 | No | ns | >0,9999 |  | |
|  | Ctrl: nacl vs. CeA-VTA: nacl | -27,6 | Yes | * | 0,0188 |  |  |
|  | Ctrl: nacl vs. CeA-VTA: c21 | 8,2 | No | ns | 0,6988 |  |  |
|  | Ctrl: c21 vs. CeA-VTA: nacl | -27,6 | Yes | * | 0,0188 |  |  |
|  | Ctrl: c21 vs. CeA-VTA: c21 | 8,2 | No | ns | 0,6988 |  |  |
|  | CeA-VTA: nacl vs. CeA-VTA: c21 | 35,8 | Yes | ** | 0,0027 |  |  |
| F2 E | **Maintenance** | **ANOVA table** | **SS** | **DF** | **MS** | **F (DFn, DFd)** | **P value** |
|  |  | Column Factor | 3318 | 3 | 1106 | F (3, 16) = 8,848 | P=0,0011 |
|  | **Holm-Šídák's multiple comparisons test** | **Predicted (LS) mean diff,** | **Below threshold?** | **Summary** | **Adjusted P Value** |  |  |
|  | Ctrl: NaCl vs. Ctrl: c21 | -0,1846 | No | ns | 0,9795 | A-B |  |
|  | Ctrl: NaCl vs. CeA-VTA: NaCl | 4,014 | No | ns | 0,9154 | A-C |  |
|  | Ctrl: NaCl vs. CeA-VTA: c21 | 30,77 | Yes | ** | 0,0028 | A-D |  |
|  | Ctrl: c21 vs. CeA-VTA: NaCl | 4,199 | No | ns | 0,9154 | B-C |  |
|  | Ctrl: c21 vs. CeA-VTA: c21 | 30,95 | Yes | ** | 0,0028 | B-D |  |
|  | CeA-VTA: NaCl vs. CeA-VTA: c21 | 26,76 | Yes | ** | 0,0065 |  |  |
| F2 F | **Blocking** | **ANOVA table** | **SS** | **DF** | **MS** | **F (DFn, DFd)** | **P value** |
|  |  | Column Factor | 450 | 3 | 150 | F (3, 16) = 6,018 | P=0,0060 |
|  | **Holm-Šídák's multiple comparisons test** | **Mean Diff,** | **Below threshold?** | **Summary** | **Adjusted P Value** |  |  |
|  | Ctrl: NaCl vs. Ctrl: c21 | 2,4 | No | ns | 0,7065 | A-B |  |
|  | Ctrl: NaCl vs. CeA-VTA: NaCl | 4,2 | No | ns | 0,4921 | A-C |  |
|  | Ctrl: NaCl vs. CeA-VTA: c21 | -8,2 | No | ns | 0,0756 | A-D |  |
|  | Ctrl: c21 vs. CeA-VTA: NaCl | 1,8 | No | ns | 0,7065 | B-C |  |
|  | Ctrl: c21 vs. CeA-VTA: c21 | -10,6 | Yes | * | 0,0199 | B-D |  |
|  | CeA-VTA: NaCl vs. CeA-VTA: c21 | -12,4 | Yes | ** | 0,0072 | C-D |  |
| F2 G |  | I CeA-VTA |  | M CeA-VTA |  | B CeA-VTA |  |
|  | One sample t test | p=0,1635 | t=1,705, df=4 | p=0,0064 | t=5,221, df=4 | p=0,0484 | t=5,221, df=4 |
| F2 I | GAD67/input | GABA+ vs. GABA- | Unpaired t test | p= 0,0018 | t=4,583, df=8 |  |  |
| F3 E | **Initiation** | **ANOVA table** | **SS** | **DF** | **MS** | **F (DFn, DFd)** | **P value** |
|  |  | Column Factor | 5264 | 5 | 1053 | F (5, 32) = 5,362 | P=0,0011 |
|  | **Holm-Šídák's multiple comparisons test** | **Mean Diff,** | **Below threshold?** | **Summary** | **Adjusted P Value** |  |  |
|  | Ctrl: NaCl vs. Ctrl:c21 | 9,667 | No | ns | 0,748 | A-B |  |
|  | Ctrl: NaCl vs. VTA-ACC: NaCl | 13,33 | No | ns | 0,5546 | A-C |  |
|  | Ctrl: NaCl vs. VTA-ACC: c21 | -17,67 | No | ns | 0,31 | A-D |  |
|  | Ctrl: NaCl vs. VTA-OFC: NaCl | 16,17 | No | ns | 0,3469 | A-E |  |
|  | Ctrl: NaCl vs. VTA-OFC: c21 | -5,69 | No | ns | 0,8793 | A-F |  |
|  | Ctrl:c21 vs. VTA-ACC: NaCl | 3,667 | No | ns | 0,8799 | B-C |  |
|  | Ctrl:c21 vs. VTA-ACC: c21 | -27,33 | Yes | * | 0,0248 | B-D |  |
|  | Ctrl:c21 vs. VTA-OFC: NaCl | 6,5 | No | ns | 0,8793 | B-E |  |
|  | Ctrl:c21 vs. VTA-OFC: c21 | -15,36 | No | ns | 0,3776 | B-F |  |
|  | VTA-ACC: NaCl vs. VTA-ACC: c21 | -31 | Yes | ** | 0,0078 | C-D |  |
|  | VTA-ACC: NaCl vs. VTA-OFC: NaCl | 2,833 | No | ns | 0,8799 | C-E |  |
|  | VTA-ACC: NaCl vs. VTA-OFC: c21 | -19,02 | No | ns | 0,2028 | C-F |  |
|  | VTA-ACC: c21 vs. VTA-OFC: NaCl | 33,83 | Yes | ** | 0,002 | D-E |  |
|  | VTA-ACC: c21 vs. VTA-OFC: c21 | 11,98 | No | ns | 0,5791 | D-F |  |
|  | VTA-OFC: NaCl vs. VTA-OFC: c21 | -21,86 | No | ns | 0,0741 | E-F |  |
| F3 F | **Maintenance** | **ANOVA table** | **SS** | **DF** | **MS** | **F (DFn, DFd)** | **P value** |
|  |  | Column Factor | 5238 | 5 | 1048 | F (5, 32) = 7,189 | P=0,0001 |
|  | **Holm-Šídák's multiple comparisons test** | **Mean Diff,** | **Below threshold?** | **Summary** | **Adjusted P Value** |  |  |
|  | Ctrl: NaCl vs. Ctrl:c21 | -5,356 | No | ns | 0,8487 | A-B |  |
|  | Ctrl: NaCl vs. VTA-ACC: NaCl | 14,15 | No | ns | 0,3403 | A-C |  |
|  | Ctrl: NaCl vs. VTA-ACC: c21 | -11,61 | No | ns | 0,4879 | A-D |  |
|  | Ctrl: NaCl vs. VTA-OFC: NaCl | 16,22 | No | ns | 0,1786 | A-E |  |
|  | Ctrl: NaCl vs. VTA-OFC: c21 | -13,17 | No | ns | 0,3447 | A-F |  |
|  | Ctrl:c21 vs. VTA-ACC: NaCl | 19,51 | No | ns | 0,0828 | B-C |  |
|  | Ctrl:c21 vs. VTA-ACC: c21 | -6,252 | No | ns | 0,8487 | B-D |  |
|  | Ctrl:c21 vs. VTA-OFC: NaCl | 21,57 | Yes | * | 0,0325 | B-E |  |
|  | Ctrl:c21 vs. VTA-OFC: c21 | -7,815 | No | ns | 0,7677 | B-F |  |
|  | VTA-ACC: NaCl vs. VTA-ACC: c21 | -25,76 | Yes | ** | 0,0097 | C-D |  |
|  | VTA-ACC: NaCl vs. VTA-OFC: NaCl | 2,066 | No | ns | 0,9426 | C-E |  |
|  | VTA-ACC: NaCl vs. VTA-OFC: c21 | -27,32 | Yes | ** | 0,0037 | C-F |  |
|  | VTA-ACC: c21 vs. VTA-OFC: NaCl | 27,83 | Yes | ** | 0,0033 | D-E |  |
|  | VTA-ACC: c21 vs. VTA-OFC: c21 | -1,563 | No | ns | 0,9426 | D-F |  |
|  | VTA-OFC: NaCl vs. VTA-OFC: c21 | -29,39 | Yes | ** | 0,0011 | E-F |  |
| F3 G | **Blocking** | **ANOVA table** | **SS** | **DF** | **MS** | **F (DFn, DFd)** | **P value** |
|  |  | Column Factor | 701,7 | 5 | 140,3 | F (5, 32) = 6,295 | P=0,0004 |
|  | **Holm-Šídák's multiple comparisons test** | **Mean Diff,** | **Below threshold?** | **Summary** | **Adjusted P Value** |  |  |
|  | Ctrl: NaCl vs. Ctrl: c21 | 1,333 | No | ns | 0,9458 | A-B |  |
|  | Ctrl: NaCl vs. VTA-ACC: NaCl | -7,833 | No | ns | 0,0693 | A-C |  |
|  | Ctrl: NaCl vs. VTA-ACC: c21 | -1,333 | No | ns | 0,9458 | A-D |  |
|  | Ctrl: NaCl vs. VTA-OFC: NaCl | -10,5 | Yes | ** | 0,0049 | A-E |  |
|  | Ctrl: NaCl vs. VTA-OFC: c21 | -2,643 | No | ns | 0,8991 | A-F |  |
|  | Ctrl: c21 vs. VTA-ACC: NaCl | -9,167 | Yes | * | 0,0239 | B-C |  |
|  | Ctrl: c21 vs. VTA-ACC: c21 | -2,667 | No | ns | 0,8991 | B-D |  |
|  | Ctrl: c21 vs. VTA-OFC: NaCl | -11,83 | Yes | ** | 0,0012 | B-E |  |
|  | Ctrl: c21 vs. VTA-OFC: c21 | -3,976 | No | ns | 0,6518 | B-F |  |
|  | VTA-ACC: NaCl vs. VTA-ACC: c21 | 6,5 | No | ns | 0,1904 | C-D |  |
|  | VTA-ACC: NaCl vs. VTA-OFC: NaCl | -2,667 | No | ns | 0,8991 | C-E |  |
|  | VTA-ACC: NaCl vs. VTA-OFC: c21 | 5,19 | No | ns | 0,3738 | C-F |  |
|  | VTA-ACC: c21 vs. VTA-OFC: NaCl | -9,167 | Yes | * | 0,0185 | D-E |  |
|  | VTA-ACC: c21 vs. VTA-OFC: c21 | -1,31 | No | ns | 0,9458 | D-F |  |
|  | VTA-OFC: NaCl vs. VTA-OFC: c21 | 7,857 | Yes | * | 0,0419 | E-F |  |
| F3 H | % test- Ctrl | I VTA-ACC | I VTA-OFC | M VTA-ACC | M VTA-OFC | B VTA-ACC | B VTA-OFC |
|  | One sample t test | p=0,0114 | p=0,0253 | p=0,2091 | p=0,1432 | p=0,1831 | p=0,0058 |
|  |  | t=3,898, df=5 | t=2,960, df=6 | t=1,441, df=5 | t=1,684, df=6 | t=1,545, df=5 | t=4,190, df=6 |
| F4 B | inputs | ACC-CeA vs OFC-CeA | Unpaired t test | 0,001 | t=6,917, df=5 |  |  |
| F4 H | **Initiation** | **ANOVA table** | **SS** | **DF** | **MS** | **F (DFn, DFd)** | **P value** |
|  |  | Column Factor | 1759 | 5 | 351,8 | F (5, 28) = 4,941 | P=0,0023 |
|  | **Holm-Šídák's multiple comparisons test** | **Mean Diff,** | **Below threshold?** | **Summary** | **Adjusted P Value** |  |  |
|  | Ctrl: NaCl vs. Ctrl: c21 | 0 | No | ns | >0,9999 | A-B |  |
|  | Ctrl: NaCl vs. ACC-CeA: NaCl | -1,1 | No | ns | 0,9952 | A-C |  |
|  | Ctrl: NaCl vs. ACC-CeA: c21 | 10,9 | No | ns | 0,3191 | A-D |  |
|  | Ctrl: NaCl vs. OFC-CeA: NaCl | -3,433 | No | ns | 0,9857 | A-E |  |
|  | Ctrl: NaCl vs. OFC-CeA: c21 | 15,73 | No | ns | 0,0583 | A-F |  |
|  | Ctrl: c21 vs. ACC-CeA: NaCl | -1,1 | No | ns | 0,9952 | B-C |  |
|  | Ctrl: c21 vs. ACC-CeA: c21 | 10,9 | No | ns | 0,3191 | B-D |  |
|  | Ctrl: c21 vs. OFC-CeA: NaCl | -3,433 | No | ns | 0,9857 | B-E |  |
|  | Ctrl: c21 vs. OFC-CeA: c21 | 15,73 | No | ns | 0,0583 | B-F |  |
|  | ACC-CeA: NaCl vs. ACC-CeA: c21 | 12 | No | ns | 0,1844 | C-D |  |
|  | ACC-CeA: NaCl vs. OFC-CeA: NaCl | -2,333 | No | ns | 0,9857 | C-E |  |
|  | ACC-CeA: NaCl vs. OFC-CeA: c21 | 16,83 | Yes | * | 0,0245 | C-F |  |
|  | ACC-CeA: c21 vs. OFC-CeA: NaCl | -14,33 | No | ns | 0,069 | D-E |  |
|  | ACC-CeA: c21 vs. OFC-CeA: c21 | 4,833 | No | ns | 0,9392 | D-F |  |
|  | OFC-CeA: NaCl vs. OFC-CeA: c21 | 19,17 | Yes | ** | 0,0075 | E-F |  |
| F4 I | **Maintenance** | **ANOVA table** | **SS** | **DF** | **MS** | **F (DFn, DFd)** | **P value** |
|  |  | Column Factor | 7835 | 5 | 1567 | F (5, 28) = 8,960 | P<0,0001 |
|  | **Holm-Šídák's multiple comparisons test** | **Mean Diff,** | **Below threshold?** | **Summary** | **Adjusted P Value** |  |  |
|  | Ctrl: NaCl vs. Ctrl: c21 | -0,1846 | No | ns | 0,9826 | A-B |  |
|  | Ctrl: NaCl vs. ACC-CeA: NaCl | 4,846 | No | ns | 0,9402 | A-C |  |
|  | Ctrl: NaCl vs. ACC-CeA: c21 | 37,33 | Yes | *** | 0,0009 | A-D |  |
|  | Ctrl: NaCl vs. OFC-CeA: NaCl | -6,403 | No | ns | 0,9402 | A-E |  |
|  | Ctrl: NaCl vs. OFC-CeA: c21 | 19,69 | No | ns | 0,1773 | A-F |  |
|  | Ctrl: c21 vs. ACC-CeA: NaCl | 5,03 | No | ns | 0,9402 | B-C |  |
|  | Ctrl: c21 vs. ACC-CeA: c21 | 37,51 | Yes | *** | 0,0009 | B-D |  |
|  | Ctrl: c21 vs. OFC-CeA: NaCl | -6,218 | No | ns | 0,9402 | B-E |  |
|  | Ctrl: c21 vs. OFC-CeA: c21 | 19,88 | No | ns | 0,1773 | B-F |  |
|  | ACC-CeA: NaCl vs. ACC-CeA: c21 | 32,48 | Yes | ** | 0,0025 | C-D |  |
|  | ACC-CeA: NaCl vs. OFC-CeA: NaCl | -11,25 | No | ns | 0,6277 | C-E |  |
|  | ACC-CeA: NaCl vs. OFC-CeA: c21 | 14,85 | No | ns | 0,3609 | C-F |  |
|  | ACC-CeA: c21 vs. OFC-CeA: NaCl | -43,73 | Yes | **** | <0,0001 | D-E |  |
|  | ACC-CeA: c21 vs. OFC-CeA: c21 | -17,64 | No | ns | 0,2064 | D-F |  |
|  | OFC-CeA: NaCl vs. OFC-CeA: c21 | 26,09 | Yes | * | 0,0213 | E-F |  |
| F4 J | **Blocking** | **ANOVA table** | **SS** | **DF** | **MS** | **F (DFn, DFd)** | **P value** |
|  |  | Column Factor | 2521 | 5 | 504,1 | F (5, 28) = 21,65 | P<0,0001 |
|  | **Holm-Šídák's multiple comparisons test** | **Mean Diff,** | **Below threshold?** | **Summary** | **Adjusted P Value** |  |  |
|  | Ctrl: NaCl vs. Ctrl: c21 | 2,4 | No | ns | 0,8533 | A-B |  |
|  | Ctrl: NaCl vs. ACC-CeA: NaCl | 1,333 | No | ns | 0,8787 | A-C |  |
|  | Ctrl: NaCl vs. ACC-CeA: c21 | -17,5 | Yes | **** | <0,0001 | A-D |  |
|  | Ctrl: NaCl vs. OFC-CeA: NaCl | 5 | No | ns | 0,5147 | A-E |  |
|  | Ctrl: NaCl vs. OFC-CeA: c21 | -13 | Yes | *** | 0,001 | A-F |  |
|  | Ctrl: c21 vs. ACC-CeA: NaCl | -1,067 | No | ns | 0,8787 | B-C |  |
|  | Ctrl: c21 vs. ACC-CeA: c21 | -19,9 | Yes | **** | <0,0001 | B-D |  |
|  | Ctrl: c21 vs. OFC-CeA: NaCl | 2,6 | No | ns | 0,8533 | B-E |  |
|  | Ctrl: c21 vs. OFC-CeA: c21 | -15,4 | Yes | *** | 0,0001 | B-F |  |
|  | ACC-CeA: NaCl vs. ACC-CeA: c21 | -18,83 | Yes | **** | <0,0001 | C-D |  |
|  | ACC-CeA: NaCl vs. OFC-CeA: NaCl | 3,667 | No | ns | 0,6699 | C-E |  |
|  | ACC-CeA: NaCl vs. OFC-CeA: c21 | -14,33 | Yes | *** | 0,0002 | C-F |  |
|  | ACC-CeA: c21 vs. OFC-CeA: NaCl | 22,5 | Yes | **** | <0,0001 | D-E |  |
|  | ACC-CeA: c21 vs. OFC-CeA: c21 | 4,5 | No | ns | 0,5276 | D-F |  |
|  | OFC-CeA: NaCl vs. OFC-CeA: c21 | -18 | Yes | **** | <0,0001 | E-F |  |
| F4 K |  | I ACC-CeA | I OFC-CeA | M ACC-CeA | MOFC-CeA | B ACC-CeA | B OFC-CeA |
| Wilcoxon Signed Rank Test | | p=0,1563 | p=0,0313 | p=0,0313 | p=0,0938 | p=0,0313 | p=0,0313 |
|  | **Title** | **group** | **test** | **p** | **t,df** |  |  |
| S1 A | Duration of social contact | Ctrl vs. SI | Unpaired t test | 0,006 | t=3,233, df=14 |  |  |
| S1 D | **NpHR** | **ANOVA table** | **SS** | **DF** | **MS** | **F (DFn, DFd)** | **P value** |
|  |  | on/off batch x group | 357,4 | 6 | 59,56 | F (6, 66) = 1,504 | P=0,1905 |
|  |  | on/off batch | 982 | 2 | 491 | F (1,460, 48,17) = 12,40 | P=0,0002 |
|  |  | group | 3789 | 3 | 1263 | F (3, 33) = 19,01 | P<0,0001 |
|  |  | Subject | 2193 | 33 | 66,45 | F (33, 66) = 1,678 | P=0,0373 |
|  | **Holm-Šídák's multiple comparisons test** | **Mean Diff,** | **Below threshold?** | **Summary** | **Adjusted P Value** |  |  |
|  | Food cells |  |  |  |  |  | |
|  | Baseline vs. on-off:1-5 | 2,586 | No | ns | 0,2975 |  |  |
|  | Baseline vs. on-off:6-10 | 7,101 | Yes | * | 0,0125 |  |  |
|  | on-off:1-5 vs. on-off:6-10 | 4,515 | Yes | *** | 0,0007 |  |  |
|  | Social cells |  |  |  |  |  |  |
|  | Baseline vs. on-off:1-5 | 9,773 | Yes | * | 0,049 |  |  |
|  | Baseline vs. on-off:6-10 | 12,86 | Yes | * | 0,0221 |  |  |
|  | on-off:1-5 vs. on-off:6-10 | 3,088 | No | ns | 0,0794 |  |  |
|  | No light: Food cells |  |  |  |  |  |  |
|  | Baseline vs. on-off:1-5 | 2,365 | No | ns | 0,8351 |  |  |
|  | Baseline vs. on-off:6-10 | 3,008 | No | ns | 0,7791 |  |  |
|  | on-off:1-5 vs. on-off:6-10 | 0,6429 | No | ns | 0,8392 |  |  |
|  | No Light: Social cells |  |  |  |  |  |  |
|  | Baseline vs. on-off:1-5 | 1,087 | No | ns | 0,5729 |  |  |
|  | Baseline vs. on-off:6-10 | 7,071 | No | ns | 0,1641 |  |  |
|  | on-off:1-5 vs. on-off:6-10 | 5,984 | No | ns | 0,1234 |  |  |
| S1 E | **cage exploration Chr2** | **ANOVA table** | **SS** | **DF** | **MS** | **F (DFn, DFd)** | **P value** |
|  |  | Row Factor x Column Factor | 921,5 | 3 | 307,2 | F (3, 26) = 5,663 | P=0,0040 |
|  |  | Row Factor | 335,7 | 1 | 335,7 | F (1, 26) = 6,189 | P=0,0196 |
|  |  | Column Factor | 2461 | 3 | 820,5 | F (3, 26) = 11,59 | P<0,0001 |
|  |  | Subject | 1840 | 26 | 70,78 | F (26, 26) = 1,305 | P=0,2510 |
|  |  | Residual | 1410 | 26 | 54,24 |  |  |
|  | **Holm-Šídák's multiple comparisons test** | **Predicted (LS) mean diff,** | **Below threshold?** | **Summary** | **Adjusted P Value** |  |  |
|  | baseline - test |  |  |  |  |  | |
|  | YL: social cells | -8,155 | No | ns | 0,0806 |  |  |
|  | YL: non-social cells | 8,038 | No | ns | 0,0806 |  |  |
|  | no YL: non-social cells | -9,732 | No | ns | 0,0787 |  |  |
|  | no YL: social cells | -9,185 | No | ns | 0,0806 |  |  |
| S1 F | **cage exploration NpHR** | **ANOVA table** | **SS** | **DF** | **MS** | **F (DFn, DFd)** | **P value** |
|  |  | time x group | 1288 | 3 | 429,2 | F (3, 31) = 6,437 | P=0,0016 |
|  |  | time | 165,8 | 1 | 165,8 | F (1, 31) = 2,487 | P=0,1250 |
|  |  | group | 2564 | 3 | 854,7 | F (3, 31) = 10,04 | P<0,0001 |
|  |  | Subject | 2639 | 31 | 85,13 | F (31, 31) = 1,277 | P=0,2502 |
|  | **Holm-Šídák's multiple comparisons test** | **Predicted (LS) mean diff,** | **Below threshold?** | **Summary** | **Adjusted P Value** |  |  |
|  | baseline - test |  |  |  |  |  | |
|  | BL: non-social cells | 14,51 | Yes | ** | 0,0028 |  |  |
|  | BL: social cells | 9,05 | Yes | * | 0,0319 |  |  |
|  | no BL: non-social cells | -7,024 | No | ns | 0,2215 |  |  |
|  | no BL: social cells | -3,918 | No | ns | 0,376 |  |  |
| S1 G | **Lever pressing** | **ANOVA table** | **SS** | **DF** | **MS** | **F (DFn, DFd)** | **P value** |
|  |  | Treatment (between columns) | 3472 | 5 | 694,5 | F (5, 114) = 34,46 | P<0,0001 |
|  | **Holm-Šídák's multiple comparisons test** | **Mean Diff,** | **Below threshold?** | **Summary** | **Adjusted P Value** |  |  |
|  | Activation: social cells vs. Activation: food cells | -2,635 | No | ns | 0,1853 | A-B |  |
|  | Activation: social cells vs. No light: social cells | -11,18 | Yes | **** | <0,0001 | A-C |  |
|  | Activation: social cells vs. No light: food cells | -7,693 | Yes | **** | <0,0001 | A-D |  |
|  | Activation: social cells vs. Inhibition: social cells | 2,205 | No | ns | 0,2311 | A-E |  |
|  | Activation: social cells vs. Inhibition: food cells | 4,048 | Yes | * | 0,0256 | A-F |  |
|  | Activation: food cells vs. No light: social cells | -8,548 | Yes | **** | <0,0001 | B-C |  |
|  | Activation: food cells vs. No light: food cells | -5,058 | Yes | ** | 0,0037 | B-D |  |
|  | Activation: food cells vs. Inhibition: social cells | 4,84 | Yes | ** | 0,0054 | B-E |  |
|  | Activation: food cells vs. Inhibition: food cells | 6,683 | Yes | **** | <0,0001 | B-F |  |
|  | No light: social cells vs. No light: food cells | 3,489 | No | ns | 0,0605 | C-D |  |
|  | No light: social cells vs. Inhibition: social cells | 13,39 | Yes | **** | <0,0001 | C-E |  |
|  | No light: social cells vs. Inhibition: food cells | 15,23 | Yes | **** | <0,0001 | C-F |  |
|  | No light: food cells vs. Inhibition: social cells | 9,898 | Yes | **** | <0,0001 | D-E |  |
|  | No light: food cells vs. Inhibition: food cells | 11,74 | Yes | **** | <0,0001 | D-F |  |
|  | Inhibition: social cells vs. Inhibition: food cells | 1,843 | No | ns | 0,2311 | E-F |  |
| S1 H | **Social approaches** | **Fixed effects- Social approaches** | **P value** | **P value summary** | **Statistically significant (P < 0,05)?** | **F (DFn, DFd)** |  |
|  |  | Row Factor | 0,8203 | ns | No | F (2, 24) = 0,1997 |  |
|  |  | Column Factor | 0,1533 | ns | No | F (1, 23) = 2,181 |  |
|  |  | Row Factor x Column Factor | 0,289 | ns | No | F (2, 23) = 1,311 |  |
| S1 H | **USVs** | **ANOVA table** | **SS** | **DF** | **MS** | **F (DFn, DFd)** | **P value** |
|  |  | group x test | 362170 | 2 | 181085 | F (2, 13) = 1,116 | P=0,3571 |
|  |  | group | 232285 | 2 | 116143 | F (2, 13) = 0,2148 | P=0,8095 |
|  |  | test | 1941906 | 1 | 1941906 | F (1, 13) = 11,96 | P=0,0042 |
|  |  | Subject | 7028095 | 13 | 540623 | F (13, 13) = 3,331 | P=0,0192 |
| S1 I | **Lever pressing** | **ANOVA table** | **SS** | **DF** | **MS** | **F (DFn, DFd)** | **P value** |
|  |  | group | 10365 | 2 | 5182 | F (2, 24) = 1,684 | P=0,2069 |

| S2-ACC social cells | ACC-GLU | | | | | |  |
| --- | --- | --- | --- | --- | --- | --- | --- |
|  | **ANOVA table** | **SS** | **DF** | **MS** | **F (DFn, DFd)** | **P value** |  |
|  | Treatment (between columns) | 0,4671 | 2 | 0,2335 | F (2, 11) = 0,1642 | P=0,8506 |  |
|  | ACC-GABA | Kruskal-Wallis test |  |  | p = 0,1535 |  |  |
|  | ACC-5HT | | | | | |  |
|  | **ANOVA table** | **SS** | **DF** | **MS** | **F (DFn, DFd)** | **P value** |  |
|  | Treatment (between columns) | 25400 | 2 | 12700 | F (2, 11) = 2,053 | P=0,1747 |  |
|  | ACC-NA | Kruskal-Wallis test |  |  | p=0,0574 |  |  |
|  | ACC-DA | Kruskal-Wallis test |  |  | p=0,0039 |  |  |
|  | **Dunn's multiple comparisons test** | **Mean rank diff,** | **Significant?** | **Summary** | **Adjusted P Value** |  |  |
|  | Ctrl vs. Activation: "Social cells" | -3,25 | No | ns | 0,8157 | A-B |  |
|  | Ctrl vs. Inhibition: "Social cells" | 4,5 | No | ns | 0,2869 | A-C |  |
|  | Activation: "Social cells" vs. Inhibition: "Social cells" | 7,75 | Yes | * | 0,0123 | B-C |  |
| S2-ACC food cells | ACC-GLU |  |  |  |  |  |  |
|  | **ANOVA table** | **SS** | **DF** | **MS** | **F (DFn, DFd)** | **P value** |  |
|  | Treatment (between columns) | 3,532 | 2 | 1,766 | F (2, 13) = 3,133 | P=0,0775 |  |
|  | ACC-GABA | Kruskal-Wallis test |  |  | p=0,0988 |  |  |
|  | ACC-5HT |  | | | | |  |
|  | **ANOVA table** | **SS** | **DF** | **MS** | **F (DFn, DFd)** | **P value** |  |
|  | Treatment (between columns) | 15199 | 2 | 7599 | F (2, 13) = 2,265 | P=0,1433 |  |
|  | ACC-NA |  | | | | |  |
|  | **ANOVA table** | **SS** | **DF** | **MS** | **F (DFn, DFd)** | **P value** |  |
|  | Treatment (between columns) | 35636 | 2 | 17818 | F (2, 13) = 8,344 | P=0,0047 |  |
|  | **Holm-Šídák's multiple comparisons test** | **Mean Diff,** | **Below threshold?** | **Summary** | **Adjusted P Value** |  |  |
|  | Ctrl vs. Activation: "Social cells" | 108,1 | Yes | ** | 0,0082 | A-B |  |
|  | Ctrl vs. Inhibition: "Social cells" | 109,9 | Yes | ** | 0,0082 | A-C |  |
|  | Activation: "Social cells" vs. Inhibition: "Social cells" | 1,792 | No | ns | 0,9475 | B-C |  |
|  | ACC-DA | Kruskal-Wallis test |  |  | 0,0595 |  |  |
| S2-OFC social cells | OFC-Glu |  | | | | |  |
|  | **ANOVA table** | **SS** | **DF** | **MS** | **F (DFn, DFd)** | **P value** |  |
|  | Treatment (between columns) | 13,02 | 2 | 6,51 | F (2, 11) = 6,258 | P=0,0153 |  |
|  | Holm-Šídák's multiple comparisons test | Mean Diff, | Below threshold? | Summary | Adjusted P Value |  |  |
|  | Ctrl vs. A | 2,325 | Yes | * | 0,0241 | A-B |  |
|  | Ctrl vs. I | 0,3599 | No | ns | 0,5955 | A-C |  |
|  | A vs. I | -1,965 | Yes | * | 0,0247 | B-C |  |
|  | OFC-GABA |  | | | | | |
|  | **ANOVA table** | **SS** | **DF** | **MS** | **F (DFn, DFd)** | **P value** |  |
|  | Treatment (between columns) | 0,5362 | 2 | 0,2681 | F (2, 11) = 4,583 | P=0,0357 |  |
|  | **Holm-Šídák's multiple comparisons test** | **Mean Diff,** | **Below threshold?** | **Summary** | **Adjusted P Value** |  |  |
|  | Ctrl vs. A | 0,1727 | No | ns | 0,3342 | A-B |  |
|  | Ctrl vs. I | 0,4592 | Yes | * | 0,0397 | A-C |  |
|  | A vs. I | 0,2864 | No | ns | 0,1786 | B-C |  |
|  | OFC-5HT |  |  |  |  |  |  |
|  | **ANOVA table** | **SS** | **DF** | **MS** | **F (DFn, DFd)** | **P value** |  |
|  | Treatment (between columns) | 3036 | 2 | 1518 | F (2, 11) = 0,2113 | P=0,8127 |  |
|  | OFC-NA |  |  |  |  |  |  |
|  | **ANOVA table** | **SS** | **DF** | **MS** | **F (DFn, DFd)** | **P value** |  |
|  | Treatment (between columns) | 86636 | 2 | 43318 | F (2, 11) = 13,59 | P=0,0011 |  |
|  | **Holm-Šídák's multiple comparisons test** | **Mean Diff,** | **Below threshold?** | **Summary** | **Adjusted P Value** |  |  |
|  | Ctrl vs. Activation: "Social cells" | 190,1 | Yes | ** | 0,0018 | A-B |  |
|  | Ctrl vs. Inhibition: "Social cells" | 30,41 | No | ns | 0,4218 | A-C |  |
|  | Activation: "Social cells" vs. Inhibition: "Social cells" | -159,7 | Yes | ** | 0,0022 | B-C |  |
|  | OFC-DA | Kruskal-Wallis test |  |  | p=0,0924 |  |  |
| S2-OFC food cells | OFC Glu |  | | | | | |
|  | **ANOVA table** | **SS** | **DF** | **MS** | **F (DFn, DFd)** | **P value** |  |
|  | Treatment (between columns) | 4,787 | 2 | 2,393 | F (2, 13) = 2,216 | P=0,1485 |  |
|  | OFC 5HT |  |  |  |  |  |  |
|  | **ANOVA table** | **SS** | **DF** | **MS** | **F (DFn, DFd)** | **P value** |  |
|  | Treatment (between columns) | 16742 | 2 | 8371 | F (2, 13) = 1,110 | P=0,3590 |  |
|  | OFC - NA |  | | | | | |
|  | **ANOVA table** | **SS** | **DF** | **MS** | **F (DFn, DFd)** | **P value** |  |
|  | Treatment (between columns) | 62863 | 2 | 31432 | F (2, 13) = 18,88 | P=0,0001 |  |
|  | **Holm-Šídák's multiple comparisons test** | **Mean Diff,** | **Below threshold?** | **Summary** | **Adjusted P Value** |  |  |
|  | Ctrl vs. Activation: "Social cells" | 158,1 | Yes | *** | 0,0001 | A-B |  |
|  | Ctrl vs. Inhibition: "Social cells" | 122,6 | Yes | *** | 0,0009 | A-C |  |
|  | Activation: "Social cells" vs. Inhibition: "Social cells" | -35,42 | No | ns | 0,1566 | B-C |  |
|  | OFC DA | Kruskal-Wallis test |  |  | p=0,5106 |  |  |
| S2 mPFC social cells | PFC Glu |  | | | | |  |
|  | **ANOVA table** | **SS** | **DF** | **MS** | **F (DFn, DFd)** | **P value** |  |
|  | Treatment (between columns) | 33,79 | 2 | 16,89 | F (2, 11) = 7,530 | P=0,0087 |  |
|  | Holm-Šídák's multiple comparisons test | Mean Diff, | Below threshold? | Summary | Adjusted P Value |  |  |
|  | Ctrl vs. A | 1,907 | No | ns | 0,1633 | A-B |  |
|  | Ctrl vs. I | -1,828 | No | ns | 0,1633 | A-C |  |
|  | A vs. I | -3,734 | Yes | ** | 0,0079 | B-C |  |
|  | PFC GABA |  |  |  |  |  |  |
|  | **ANOVA table** | **SS** | **DF** | **MS** | **F (DFn, DFd)** | **P value** |  |
|  | Treatment (between columns) | 0,01902 | 2 | 0,009509 | F (2, 11) = 0,1702 | P=0,8456 |  |
|  | PFC 5HT |  |  |  |  |  |  |
|  | **ANOVA table** | **SS** | **DF** | **MS** | **F (DFn, DFd)** | **P value** |  |
|  | Treatment (between columns) | 25111 | 2 | 12555 | F (2, 11) = 1,217 | P=0,3330 |  |
|  | PFC NA |  | | | | |  |
|  | **ANOVA table** | **SS** | **DF** | **MS** | **F (DFn, DFd)** | **P value** |  |
|  | Treatment (between columns) | 58828 | 2 | 29414 | F (2, 11) = 5,326 | P=0,0241 |  |
|  | **Holm-Šídák's multiple comparisons test** | **Mean Diff,** | **Below threshold?** | **Summary** | **Adjusted P Value** |  |  |
|  | Ctrl vs. Activation: "Social cells" | 171,5 | Yes | * | 0,0225 | A-B |  |
|  | Ctrl vs. Inhibition: "Social cells" | 83,99 | No | ns | 0,1817 | A-C |  |
|  | Activation: "Social cells" vs. Inhibition: "Social cells" | -87,5 | No | ns | 0,1817 | B-C |  |
|  | PFC DA | Kruskal-Wallis test |  |  | 0,9479 |  |  |
| S2 mPFC food cells | PFC Glu |  | | | | |  |
|  | **ANOVA table** | **SS** | **DF** | **MS** | **F (DFn, DFd)** | **P value** |  |
|  | Treatment (between columns) | 0,5211 | 2 | 0,2606 | F (2, 13) = 0,1837 | P=0,8343 |  |
|  | PFC GABA |  |  |  |  |  |  |
|  | **ANOVA table** | **SS** | **DF** | **MS** | **F (DFn, DFd)** | **P value** |  |
|  | Treatment (between columns) | 0,06131 | 2 | 0,03065 | F (2, 13) = 0,2864 | P=0,7556 |  |
|  | PFC 5HT |  |  |  |  |  |  |
|  | **ANOVA table** | **SS** | **DF** | **MS** | **F (DFn, DFd)** | **P value** |  |
|  | Treatment (between columns) | 16615 | 2 | 8308 | F (2, 13) = 1,130 | P=0,3528 |  |
|  | PFC NA |  |  |  |  |  |  |
|  | **ANOVA table** | **SS** | **DF** | **MS** | **F (DFn, DFd)** | **P value** |  |
|  | Treatment (between columns) | 58691 | 2 | 29345 | F (2, 13) = 6,979 | P=0,0087 |  |
|  | Holm-Šídák's multiple comparisons test | Mean Diff, | Below threshold? | Summary | Adjusted P Value |  |  |
|  | Ctrl vs. Activation: "Social cells" | 103,1 | No | ns | 0,0562 | A-B |  |
|  | Ctrl vs. Inhibition: "Social cells" | 155,9 | Yes | ** | 0,0076 | A-C |  |
|  | Activation: "Social cells" vs. Inhibition: "Social cells" | 52,81 | No | ns | 0,1819 | B-C |  |
|  | PFC DA | Kruskal-Wallis test |  |  | p=0,1238 |  |  |
| S2 Nacc social cells | Nacc Glu |  | | | | |  |
|  | **ANOVA table** | **SS** | **DF** | **MS** | **F (DFn, DFd)** | **P value** |  |
|  | Treatment (between columns) | 1,34 | 2 | 0,67 | F (2, 11) = 0,3911 | P=0,6854 |  |
|  | Nacc GABA | Kruskal-Wallis test |  |  | p=0,0463 |  |  |
|  | Dunn's multiple comparisons test | Mean rank diff, | Significant? | Summary | Adjusted P Value |  |  |
|  | Ctrl vs. A | -4,25 | No | ns | 0,4524 | A-B |  |
|  | Ctrl vs. I | 2,25 | No | ns | >0,9999 | A-C |  |
|  | A vs. I | 6,5 | Yes | * | 0,0482 | B-C |  |
|  | Nacc 5HT | Kruskal-Wallis test |  |  | p=0,0215 |  |  |
|  | Dunn's multiple comparisons test | Mean rank diff, | Significant? | Summary | Adjusted P Value |  |  |
|  | Ctrl vs. Social cells | -7,25 | Yes | * | 0,0427 | A-B |  |
|  | Ctrl vs. Inhibition: "Social cells" | -1,583 | No | ns | >0,9999 | A-C |  |
|  | Social cells vs. Inhibition: "Social cells" | 5,667 | No | ns | 0,1076 | B-C |  |
|  | Nacc NA | Kruskal-Wallis test |  |  | p=0,1031 |  |  |
|  | Nacc DA | Kruskal-Wallis test |  |  | p=0,1782 |  |  |
| S2 Nacc food cells | Nacc Glu |  |  |  |  |  |  |
|  | **ANOVA table** | **SS** | **DF** | **MS** | **F (DFn, DFd)** | **P value** |  |
|  | Treatment (between columns) | 3,335 | 2 | 1,667 | F (2, 13) = 0,8591 | P=0,4462 |  |
|  | Nacc GABA | Kruskal-Wallis test |  |  | p=0,3751 |  |  |
|  | Nacc 5HT |  |  |  |  |  |  |
|  | **ANOVA table** | **SS** | **DF** | **MS** | **F (DFn, DFd)** | **P value** |  |
|  | Treatment (between columns) | 972677 | 2 | 486338 | F (2, 13) = 4,560 | P=0,0316 |  |
|  | Holm-Šídák's multiple comparisons test | Mean Diff, | Below threshold? | Summary | Adjusted P Value |  |  |
|  | Ctrl vs. Social cells | -531,7 | No | ns | 0,063 | A-B |  |
|  | Ctrl vs. Inhibition: "Social cells" | -38,97 | No | ns | 0,8562 | A-C |  |
|  | Social cells vs. Inhibition: "Social cells" | 492,8 | No | ns | 0,063 | B-C |  |
|  | Nacc NA |  |  |  |  |  |  |
|  | **ANOVA table** | **SS** | **DF** | **MS** | **F (DFn, DFd)** | **P value** |  |
|  | Treatment (between columns) | 290308 | 2 | 145154 | F (2, 13) = 1,679 | P=0,2246 |  |
|  | Nacc DA |  |  |  |  |  |  |
|  | **ANOVA table** | **SS** | **DF** | **MS** | **F (DFn, DFd)** | **P value** |  |
|  | Treatment (between columns) | 6680556 | 2 | 3340278 | F (2, 13) = 0,7639 | P=0,4857 |  |
| S2 VTA social cells | VTA Glu |  |  |  |  |  |  |
|  | **ANOVA table** | **SS** | **DF** | **MS** | **F (DFn, DFd)** | **P value** |  |
|  | Treatment (between columns) | 0,2955 | 2 | 0,1477 | F (2, 11) = 0,3730 | P=0,6971 |  |
|  | VTA GABA |  |  |  |  |  |  |
|  | **ANOVA table** | **SS** | **DF** | **MS** | **F (DFn, DFd)** | **P value** |  |
|  | Treatment (between columns) | 13,38 | 2 | 6,69 | F (2, 11) = 19,91 | P=0,0002 |  |
|  | Holm-Šídák's multiple comparisons test | Mean Diff, | Below threshold? | Summary | Adjusted P Value |  |  |
|  | Ctrl vs. A | 2,287 | Yes | *** | 0,0005 | A-B |  |
|  | Ctrl vs. I | 0,2206 | No | ns | 0,5674 | A-C |  |
|  | A vs. I | -2,066 | Yes | *** | 0,0005 | B-C |  |
|  | VTA 5HT |  |  |  |  |  |  |
|  | **ANOVA table** | **SS** | **DF** | **MS** | **F (DFn, DFd)** | **P value** |  |
|  | Treatment (between columns) | 533153 | 2 | 266577 | F (2, 11) = 4,189 | P=0,0444 |  |
|  | VTA NA |  |  |  |  |  |  |
|  | **ANOVA table** | **SS** | **DF** | **MS** | **F (DFn, DFd)** | **P value** |  |
|  | Treatment (between columns) | 2730 | 2 | 1365 | F (2, 11) = 0,1598 | P=0,8543 |  |
|  | VTA DA |  |  |  |  |  |  |
|  | **ANOVA table** | **SS** | **DF** | **MS** | **F (DFn, DFd)** | **P value** |  |
|  | Treatment (between columns) | 437958 | 2 | 218979 | F (2, 11) = 0,5138 | P=0,6119 |  |
| S2 VTA food cells | VTA Glu |  |  |  |  |  |  |
|  | **ANOVA table** | **SS** | **DF** | **MS** | **F (DFn, DFd)** | **P value** |  |
|  | Treatment (between columns) | 1,662 | 2 | 0,8309 | F (2, 13) = 0,9488 | P=0,4125 |  |
|  | VTA GABA |  |  |  |  |  |  |
|  | **ANOVA table** | **SS** | **DF** | **MS** | **F (DFn, DFd)** | **P value** |  |
|  | Treatment (between columns) | 1,148 | 2 | 0,5738 | F (2, 13) = 0,5535 | P=0,5879 |  |
|  | VTA 5HT |  |  |  |  |  |  |
|  | **ANOVA table** | **SS** | **DF** | **MS** | **F (DFn, DFd)** | **P value** |  |
|  | Treatment (between columns) | 662023 | 2 | 331011 | F (2, 13) = 3,305 | P=0,0691 |  |
|  | VTA NA |  |  |  |  |  |  |
|  | **ANOVA table** | **SS** | **DF** | **MS** | **F (DFn, DFd)** | **P value** |  |
|  | Treatment (between columns) | 47720 | 2 | 23860 | F (2, 13) = 1,329 | P=0,2985 |  |
|  | VTA DA | Kruskal-Wallis test |  |  | p=0,0775 |  |  |
| S2 social cells Hipp | Hipp Glu | Kruskal-Wallis test |  |  | p=0,4080 |  |  |
|  | Hipp GABA |  | | | | |  |
|  | **ANOVA table** | **SS** | **DF** | **MS** | **F (DFn, DFd)** | **P value** |  |
|  | Treatment (between columns) | 1,744 | 2 | 0,8721 | F (2, 11) = 2,087 | P=0,1704 |  |
|  | Hipp 5HT |  |  |  |  |  |  |
|  | **ANOVA table** | **SS** | **DF** | **MS** | **F (DFn, DFd)** | **P value** |  |
|  | Treatment (between columns) | 50760 | 2 | 25380 | F (2, 11) = 2,504 | P=0,1270 |  |
|  | Hipp NA |  |  |  |  |  |  |
|  | **ANOVA table** | **SS** | **DF** | **MS** | **F (DFn, DFd)** | **P value** |  |
|  | Treatment (between columns) | 64013 | 2 | 32007 | F (2, 11) = 3,793 | P=0,0559 |  |
|  | Hipp DA | Kruskal-Wallis test |  |  | p=0,2159 |  |  |
| S2 food cells Hipp | Hipp Glu |  |  |  |  |  |  |
|  | **ANOVA table** | **SS** | **DF** | **MS** | **F (DFn, DFd)** | **P value** |  |
|  | Treatment (between columns) | 11,96 | 2 | 5,98 | F (2, 13) = 10,42 | P=0,0020 |  |
|  | Holm-Šídák's multiple comparisons test | Mean Diff, | Below threshold? | Summary | Adjusted P Value |  |  |
|  | Ctrl vs. A | -1,62 | Yes | * | 0,0112 | A-B |  |
|  | Ctrl vs. I | 0,2571 | No | ns | 0,608 | A-C |  |
|  | A vs. I | 1,877 | Yes | ** | 0,0026 | B-C |  |
|  | Hipp GABA |  |  |  |  |  |  |
|  | **ANOVA table** | **SS** | **DF** | **MS** | **F (DFn, DFd)** | **P value** |  |
|  | Treatment (between columns) | 1,475 | 2 | 0,7374 | F (2, 13) = 2,791 | P=0,0981 |  |
|  | Residual (within columns) | 3,435 | 13 | 0,2642 |  |  |  |
|  | Hipp 5HT |  |  |  |  |  |  |
|  | ANOVA table | SS | DF | MS | F (DFn, DFd) | P value |  |
|  | Treatment (between columns) | 43177 | 2 | 21589 | F (2, 13) = 1,836 | P=0,1985 |  |
|  | Hipp NA | Kruskal-Wallis test |  |  | p=0,0023 |  |  |
|  | **Dunn's multiple comparisons test** | **Mean rank diff,** | **Significant?** | **Summary** | **Adjusted P Value** |  |  |
|  | Ctrl vs. Activation: "Social cells" | 9 | Yes | * | 0,0102 | A-B |  |
|  | Ctrl vs. Inhibition: "Social cells" | 3 | No | ns | 0,9869 | A-C |  |
|  | Activation: "Social cells" vs. Inhibition: "Social cells" | -6 | No | ns | 0,0871 | B-C |  |
|  | Hipp DA | Kruskal-Wallis test |  |  | p=0,3927 |  |  |
| S2 social cells BLA | BLA Glu |  |  |  |  |  |  |
|  | **ANOVA table** | **SS** | **DF** | **MS** | **F (DFn, DFd)** | **P value** |  |
|  | Treatment (between columns) | 4,229 | 2 | 2,114 | F (2, 11) = 1,344 | P=0,3004 |  |
|  | BLA GABA |  |  |  |  |  |  |
|  | **ANOVA table** | **SS** | **DF** | **MS** | **F (DFn, DFd)** | **P value** |  |
|  | Treatment (between columns) | 3,793 | 2 | 1,897 | F (2, 11) = 10,75 | P=0,0026 |  |
|  | Holm-Šídák's multiple comparisons test | Mean Diff, | Below threshold? | Summary | Adjusted P Value |  |  |
|  | Ctrl vs. A | 0,9343 | Yes | * | 0,0185 | A-B |  |
|  | Ctrl vs. I | 1,24 | Yes | ** | 0,0024 | A-C |  |
|  | A vs. I | 0,3056 | No | ns | 0,2836 | B-C |  |
|  | BLA 5HT |  |  |  |  |  |  |
|  | **ANOVA table** | **SS** | **DF** | **MS** | **F (DFn, DFd)** | **P value** |  |
|  | Treatment (between columns) | 8830 | 2 | 4415 | F (2, 11) = 0,2555 | P=0,7790 |  |
|  | BLA NA |  |  |  |  |  |  |
|  | **ANOVA table** | **SS** | **DF** | **MS** | **F (DFn, DFd)** | **P value** |  |
|  | Treatment (between columns) | 70059 | 2 | 35029 | F (2, 11) = 10,45 | P=0,0029 |  |
|  | Holm-Šídák's multiple comparisons test | Mean Diff, | Below threshold? | Summary | Adjusted P Value |  |  |
|  | Ctrl vs. Activation: "Social cells" | 186,4 | Yes | ** | 0,0025 | A-B |  |
|  | Ctrl vs. Inhibition: "Social cells" | 79,95 | No | ns | 0,0557 | A-C |  |
|  | Activation: "Social cells" vs. Inhibition: "Social cells" | -106,4 | Yes | * | 0,0315 | B-C |  |
|  | BLA DA |  |  |  |  |  |  |
|  | **ANOVA table** | **SS** | **DF** | **MS** | **F (DFn, DFd)** | **P value** |  |
|  | Treatment (between columns) | 509864 | 2 | 254932 | F (2, 11) = 0,6640 | P=0,5342 |  |
| S2 food cells BLA | BLA Glu |  |  |  |  |  |  |
|  | **ANOVA table** | **SS** | **DF** | **MS** | **F (DFn, DFd)** | **P value** |  |
|  | Treatment (between columns) | 0,8056 | 2 | 0,4028 | F (2, 13) = 0,5516 | P=0,5889 |  |
|  | BLA GABA | Kruskal-Wallis test |  |  | p=0,0352 |  |  |
|  | Dunn's multiple comparisons test | Mean rank diff, | Significant? | Summary | Adjusted P Value |  |  |
|  | Ctrl vs. A | 3,167 | No | ns | 0,9084 | A-B |  |
|  | Ctrl vs. I | 7,5 | Yes | * | 0,044 | A-C |  |
|  | A vs. I | 4,333 | No | ns | 0,3447 | B-C |  |
|  | BLA 5HT |  |  |  |  |  |  |
|  | **ANOVA table** | **SS** | **DF** | **MS** | **F (DFn, DFd)** | **P value** |  |
|  | Treatment (between columns) | 70222 | 2 | 35111 | F (2, 13) = 2,388 | P=0,1308 |  |
|  | BLA NA |  |  |  |  |  |  |
|  | **ANOVA table** | **SS** | **DF** | **MS** | **F (DFn, DFd)** | **P value** |  |
|  | Treatment (between columns) | 49278 | 2 | 24639 | F (2, 13) = 17,06 | P=0,0002 |  |
|  | Holm-Šídák's multiple comparisons test | Mean Diff, | Below threshold? | Summary | Adjusted P Value |  |  |
|  | Ctrl vs. Activation: "Social cells" | 127,3 | Yes | *** | 0,0005 | A-B |  |
|  | Ctrl vs. Inhibition: "Social cells" | 129 | Yes | *** | 0,0005 | A-C |  |
|  | Activation: "Social cells" vs. Inhibition: "Social cells" | 1,727 | No | ns | 0,9384 | B-C |  |
|  | BLA DA |  |  |  |  |  |  |
|  | **ANOVA table** | **SS** | **DF** | **MS** | **F (DFn, DFd)** | **P value** |  |
|  | Treatment (between columns) | 371875 | 2 | 185938 | F (2, 13) = 0,4512 | P=0,6465 |  |
| S3 E | **Initiation** | **ANOVA table** | **SS** | **DF** | **MS** | **F (DFn, DFd)** | **P value** |
|  |  | Time x Column Factor | 1769 | 3 | 589,8 | F (3, 16) = 5,865 | P=0,0067 |
|  |  | Time | 156 | 1 | 156 | F (1, 16) = 1,552 | P=0,2308 |
|  |  | Column Factor | 2705 | 3 | 901,8 | F (3, 16) = 6,735 | P=0,0038 |
|  |  | Subject | 2142 | 16 | 133,9 | F (16, 16) = 1,331 | P=0,2868 |
|  | **Holm-Šídák's multiple comparisons test** | **Mean Diff,** | **Below threshold?** | **Summary** | **Adjusted P Value** |  |  |
|  | Baseline |  |  |  |  |  | |
|  | Ctrl: NaCl vs. Ctrl: c21 | 0 | No | ns | >0,9999 |  |  |
|  | Ctrl: NaCl vs. CeA›VTA: NaCl | -11,8 | No | ns | 0,3277 |  |  |
|  | Ctrl: NaCl vs. CeA›VTA: c21 | -13,4 | No | ns | 0,3063 |  |  |
|  | Ctrl: c21 vs. CeA›VTA: NaCl | -11,8 | No | ns | 0,3277 |  |  |
|  | Ctrl: c21 vs. CeA›VTA: c21 | -13,4 | No | ns | 0,3063 |  |  |
|  | CeA›VTA: NaCl vs. CeA›VTA: c21 | -1,6 | No | ns | 0,9664 |  |  |
|  | Test |  |  |  |  |  |  |
|  | Ctrl: NaCl vs. Ctrl: c21 | 0 | No | ns | >0,9999 |  |  |
|  | Ctrl: NaCl vs. CeA›VTA: NaCl | -27,6 | Yes | ** | 0,0016 |  |  |
|  | Ctrl: NaCl vs. CeA›VTA: c21 | 8,2 | No | ns | 0,5609 |  |  |
|  | Ctrl: c21 vs. CeA›VTA: NaCl | -27,6 | Yes | ** | 0,0016 |  |  |
|  | Ctrl: c21 vs. CeA›VTA: c21 | 8,2 | No | ns | 0,5609 |  |  |
|  | CeA›VTA: NaCl vs. CeA›VTA: c21 | 35,8 | Yes | **** | <0,0001 |  |  |
|  | **Holm-Šídák's multiple comparisons test** | **Mean Diff,** | **Below threshold?** | **Summary** | **Adjusted P Value** |  |  |
|  | Baseline - Test |  |  |  |  |  |  |
|  | Ctrl: NaCl | -5,4 | No | ns | 0,6485 |  |  |
|  | Ctrl: c21 | -5,4 | No | ns | 0,6485 |  |  |
|  | CeA›VTA: NaCl | -21,2 | Yes | * | 0,0164 |  |  |
|  | CeA›VTA: c21 | 16,2 | No | ns | 0,0623 |  |  |
| S3 E | **Maintenance** | **ANOVA table** | **SS** | **DF** | **MS** | **F (DFn, DFd)** | **P value** |
|  |  | Time x Column Factor | 1012 | 3 | 337,3 | F (3, 16) = 2,965 | P=0,0635 |
|  |  | Time | 649,1 | 1 | 649,1 | F (1, 16) = 5,705 | P=0,0296 |
|  |  | Column Factor | 2596 | 3 | 865,2 | F (3, 16) = 5,900 | P=0,0065 |
|  |  | Subject | 2346 | 16 | 146,6 | F (16, 16) = 1,289 | P=0,3089 |
|  | **Holm-Šídák's multiple comparisons test** | **Mean Diff,** | **Below threshold?** | **Summary** | **Adjusted P Value** |  |  |
|  | Baseline |  |  |  |  |  | |
|  | Ctrl: NaCl vs. Ctrl: c21 | -5,258 | No | ns | 0,9169 |  |  |
|  | Ctrl: NaCl vs. CeA›VTA: NaCl | 1,431 | No | ns | 0,9169 |  |  |
|  | Ctrl: NaCl vs. CeA›VTA: c21 | 5,36 | No | ns | 0,9169 |  |  |
|  | Ctrl: c21 vs. CeA›VTA: NaCl | 6,689 | No | ns | 0,8934 |  |  |
|  | Ctrl: c21 vs. CeA›VTA: c21 | 10,62 | No | ns | 0,6254 |  |  |
|  | CeA›VTA: NaCl vs. CeA›VTA: c21 | 3,929 | No | ns | 0,9169 |  |  |
|  | Test |  |  |  |  |  |  |
|  | Ctrl: NaCl vs. Ctrl: c21 | -0,1846 | No | ns | 0,9798 |  |  |
|  | Ctrl: NaCl vs. CeA›VTA: NaCl | 4,014 | No | ns | 0,9176 |  |  |
|  | Ctrl: NaCl vs. CeA›VTA: c21 | 30,77 | Yes | *** | 0,0009 |  |  |
|  | Ctrl: c21 vs. CeA›VTA: NaCl | 4,199 | No | ns | 0,9176 |  |  |
|  | Ctrl: c21 vs. CeA›VTA: c21 | 30,95 | Yes | *** | 0,0009 |  |  |
|  | CeA›VTA: NaCl vs. CeA›VTA: c21 | 26,76 | Yes | ** | 0,0032 |  |  |
|  | **Holm-Šídák's multiple comparisons test** | **Mean Diff,** | **Below threshold?** | **Summary** | **Adjusted P Value** |  |  |
|  | Baseline - Test |  |  |  |  |  | |
|  | Ctrl: NaCl | -0,2103 | No | ns | 0,9755 |  |  |
|  | Ctrl: c21 | 4,863 | No | ns | 0,8605 |  |  |
|  | CeA›VTA: NaCl | 2,373 | No | ns | 0,9269 |  |  |
|  | CeA›VTA: c21 | 25,2 | Yes | ** | 0,0072 |  |  |
| S3 F | **CeA-VTA** | **ANOVA table** | **SS** | **DF** | **MS** | **F (DFn, DFd)** | **P value** |
|  |  | Row Factor x Column Factor | 8491143 | 1 | 8491143 | F (1, 8) = 0,1643 | P=0,6959 |
|  |  | Row Factor | 101085322 | 1 | 101085322 | F (1, 8) = 1,955 | P=0,1995 |
|  |  | Column Factor | 12751837 | 1 | 12751837 | F (1, 8) = 0,1827 | P=0,6803 |
|  |  | Subject | 558416964 | 8 | 69802120 | F (8, 8) = 1,350 | P=0,3406 |
| S3 I | **Initiation** | **Source of Variation** | **% of total variation** | **P value** | **P value summary** | **Significant?** |  |
|  |  | group x Time | 19,2 | 0,0015 | ** | Yes |  |
|  |  | group | 0,5619 | 0,3932 | ns | No |  |
|  |  | Time | 17,3 | 0,0308 | * | Yes |  |
|  |  | Subject | 38,88 | 0,089 | ns | No |  |
|  | **Holm-Šídák's multiple comparisons test** | **Predicted (LS) mean diff,** | **Below threshold?** | **Summary** | **Adjusted P Value** |  |  |
|  | Baseline |  |  |  |  |  | |
|  | Ctrl: NaCl vs. Ctrl: c21 | 8,167 | No | ns | 0,9833 |  |  |
|  | Ctrl: NaCl vs. VTA›ACC: NaCl | 7,833 | No | ns | 0,9833 |  |  |
|  | Ctrl: NaCl vs. VTA›ACC: c21 | 8 | No | ns | 0,9833 |  |  |
|  | Ctrl: NaCl vs. VTA›OFC: NaCl | 5,429 | No | ns | 0,9979 |  |  |
|  | Ctrl: NaCl vs. VTA›OFC: c21 | 8,571 | No | ns | 0,973 |  |  |
|  | Ctrl: c21 vs. VTA›ACC: NaCl | -0,3333 | No | ns | >0,9999 |  |  |
|  | Ctrl: c21 vs. VTA›ACC: c21 | -0,1667 | No | ns | >0,9999 |  |  |
|  | Ctrl: c21 vs. VTA›OFC: NaCl | -2,738 | No | ns | >0,9999 |  |  |
|  | Ctrl: c21 vs. VTA›OFC: c21 | 0,4048 | No | ns | >0,9999 |  |  |
|  | VTA›ACC: NaCl vs. VTA›ACC: c21 | 0,1667 | No | ns | >0,9999 |  |  |
|  | VTA›ACC: NaCl vs. VTA›OFC: NaCl | -2,405 | No | ns | >0,9999 |  |  |
|  | VTA›ACC: NaCl vs. VTA›OFC: c21 | 0,7381 | No | ns | >0,9999 |  |  |
|  | VTA›ACC: c21 vs. VTA›OFC: NaCl | -2,571 | No | ns | >0,9999 |  |  |
|  | VTA›ACC: c21 vs. VTA›OFC: c21 | 0,5714 | No | ns | >0,9999 |  |  |
|  | VTA›OFC: NaCl vs. VTA›OFC: c21 | 3,143 | No | ns | >0,9999 |  |  |
|  | Test |  |  |  |  |  |  |
|  | Ctrl: NaCl vs. Ctrl: c21 | 9,667 | No | ns | 0,6231 |  |  |
|  | Ctrl: NaCl vs. VTA›ACC: NaCl | 13,33 | No | ns | 0,3726 |  |  |
|  | Ctrl: NaCl vs. VTA›ACC: c21 | -17,67 | No | ns | 0,1425 |  |  |
|  | Ctrl: NaCl vs. VTA›OFC: NaCl | 16,17 | No | ns | 0,1734 |  |  |
|  | Ctrl: NaCl vs. VTA›OFC: c21 | -5,69 | No | ns | 0,8156 |  |  |
|  | Ctrl: c21 vs. VTA›ACC: NaCl | 3,667 | No | ns | 0,8452 |  |  |
|  | Ctrl: c21 vs. VTA›ACC: c21 | -27,33 | Yes | ** | 0,0035 |  |  |
|  | Ctrl: c21 vs. VTA›OFC: NaCl | 6,5 | No | ns | 0,8156 |  |  |
|  | Ctrl: c21 vs. VTA›OFC: c21 | -15,36 | No | ns | 0,2029 |  |  |
|  | VTA›ACC: NaCl vs. VTA›ACC: c21 | -31 | Yes | *** | 0,0006 |  |  |
|  | VTA›ACC: NaCl vs. VTA›OFC: NaCl | 2,833 | No | ns | 0,8452 |  |  |
|  | VTA›ACC: NaCl vs. VTA›OFC: c21 | -19,02 | No | ns | 0,0745 |  |  |
|  | VTA›ACC: c21 vs. VTA›OFC: NaCl | 33,83 | Yes | **** | <0,0001 |  |  |
|  | VTA›ACC: c21 vs. VTA›OFC: c21 | 11,98 | No | ns | 0,4101 |  |  |
|  | VTA›OFC: NaCl vs. VTA›OFC: c21 | -21,86 | Yes | * |  |  |  |
|  | **Holm-Šídák's multiple comparisons test** | **Predicted (LS) mean diff,** | **Below threshold?** | **Summary** | **Adjusted P Value** |  |  |
|  | Baseline - Test |  |  |  |  |  | |
|  | Ctrl: NaCl | 5,833 | No | ns | 0,4299 |  |  |
|  | Ctrl: c21 | 7,333 | No | ns | 0,4299 |  |  |
|  | VTA›ACC: NaCl | 11,33 | No | ns | 0,2726 |  |  |
|  | VTA›ACC: c21 | -19,83 | Yes | * | 0,0183 |  |  |
|  | VTA›OFC: NaCl | 16,57 | Yes | * | 0,0338 |  |  |
|  | VTA›OFC: c21 | -8,429 | No | ns | 0,3885 |  |  |
| S3 I | **Maintenance** | **ANOVA table** | **SS** | **DF** | **MS** | **F (DFn, DFd)** | **P value** |
|  |  | group x Time | 2859 | 5 | 571,8 | F (5, 32) = 3,469 | P=0,0129 |
|  |  | group | 98,5 | 1 | 98,5 | F (1, 32) = 0,5975 | P=0,4452 |
|  |  | Time | 3580 | 5 | 716 | F (5, 32) = 5,969 | P=0,0005 |
|  |  | Subject | 3838 | 32 | 120 | F (32, 32) = 0,7277 | P=0,8133 |
|  | **Holm-Šídák's multiple comparisons test** | **Predicted (LS) mean diff,** | **Below threshold?** | **Summary** | **Adjusted P Value** |  |  |
|  | Baseline |  |  |  |  |  | |
|  | Ctrl: NaCl vs. Ctrl: c21 | -5,81 | No | ns | 0,9581 |  |  |
|  | Ctrl: NaCl vs. VTA›ACC: NaCl | -15,61 | No | ns | 0,335 |  |  |
|  | Ctrl: NaCl vs. VTA›ACC: c21 | -14,02 | No | ns | 0,483 |  |  |
|  | Ctrl: NaCl vs. VTA›OFC: NaCl | -2,228 | No | ns | 0,9817 |  |  |
|  | Ctrl: NaCl vs. VTA›OFC: c21 | -8,055 | No | ns | 0,9262 |  |  |
|  | Ctrl: c21 vs. VTA›ACC: NaCl | -9,802 | No | ns | 0,8524 |  |  |
|  | Ctrl: c21 vs. VTA›ACC: c21 | -8,208 | No | ns | 0,9262 |  |  |
|  | Ctrl: c21 vs. VTA›OFC: NaCl | 3,582 | No | ns | 0,9721 |  |  |
|  | Ctrl: c21 vs. VTA›OFC: c21 | -2,245 | No | ns | 0,9817 |  |  |
|  | VTA›ACC: NaCl vs. VTA›ACC: c21 | 1,594 | No | ns | 0,9817 |  |  |
|  | VTA›ACC: NaCl vs. VTA›OFC: NaCl | 13,38 | No | ns | 0,483 |  |  |
|  | VTA›ACC: NaCl vs. VTA›OFC: c21 | 7,557 | No | ns | 0,9262 |  |  |
|  | VTA›ACC: c21 vs. VTA›OFC: NaCl | 11,79 | No | ns | 0,6347 |  |  |
|  | VTA›ACC: c21 vs. VTA›OFC: c21 | 5,963 | No | ns | 0,9581 |  |  |
|  | VTA›OFC: NaCl vs. VTA›OFC: c21 | -5,827 | No | ns | 0,9581 |  |  |
|  | Test |  |  |  |  |  |  |
|  | Ctrl: NaCl vs. Ctrl: c21 | -5,356 | No | ns | 0,84 |  |  |
|  | Ctrl: NaCl vs. VTA›ACC: NaCl | 14,15 | No | ns | 0,3026 |  |  |
|  | Ctrl: NaCl vs. VTA›ACC: c21 | -11,61 | No | ns | 0,4574 |  |  |
|  | Ctrl: NaCl vs. VTA›OFC: NaCl | 16,22 | No | ns | 0,1457 |  |  |
|  | Ctrl: NaCl vs. VTA›OFC: c21 | -13,17 | No | ns | 0,3096 |  |  |
|  | Ctrl: c21 vs. VTA›ACC: NaCl | 19,51 | No | ns | 0,0601 |  |  |
|  | Ctrl: c21 vs. VTA›ACC: c21 | -6,252 | No | ns | 0,84 |  |  |
|  | Ctrl: c21 vs. VTA›OFC: NaCl | 21,57 | Yes | * | 0,0201 |  |  |
|  | Ctrl: c21 vs. VTA›OFC: c21 | -7,815 | No | ns | 0,7522 |  |  |
|  | VTA›ACC: NaCl vs. VTA›ACC: c21 | -25,76 | Yes | ** | 0,0047 |  |  |
|  | VTA›ACC: NaCl vs. VTA›OFC: NaCl | 2,066 | No | ns | 0,9408 |  |  |
|  | VTA›ACC: NaCl vs. VTA›OFC: c21 | -27,32 | Yes | ** | 0,0015 |  |  |
|  | VTA›ACC: c21 vs. VTA›OFC: NaCl | 27,83 | Yes | ** | 0,0012 |  |  |
|  | VTA›ACC: c21 vs. VTA›OFC: c21 | -1,563 | No | ns | 0,9408 |  |  |
|  | VTA›OFC: NaCl vs. VTA›OFC: c21 | -29,39 | Yes | *** |  |  |  |
|  | **Holm-Šídák's multiple comparisons test** | **Predicted (LS) mean diff,** | **Below threshold?** | **Summary** | **Adjusted P Value** |  |  |
|  | Baseline - Test |  |  |  |  |  |  |
|  | Ctrl: NaCl | -5,377 | No | ns | 0,8541 |  |  |
|  | Ctrl: c21 | -4,923 | No | ns | 0,8541 |  |  |
|  | VTA›ACC: NaCl | 24,39 | Yes | * | 0,0146 |  |  |
|  | VTA›ACC: c21 | -2,967 | No | ns | 0,8541 |  |  |
|  | VTA›OFC: NaCl | 13,07 | No | ns | 0,2888 |  |  |
|  | VTA›OFC: c21 | -10,49 | No | ns | 0,443 |  |  |
| S3 J | **CtrlThCre** | **ANOVA table** | **SS** | **DF** | **MS** | **F (DFn, DFd)** | **P value** |
|  |  | Row Factor x Column Factor | 103,8 | 1 | 103,8 | F (1, 10) = 1,103e-006 | P=0,9992 |
|  |  | Row Factor | 1215692121 | 1 | 1215692121 | F (1, 10) = 12,92 | P=0,0049 |
|  |  | Column Factor | 83570243 | 1 | 83570243 | F (1, 10) = 1,593 | P=0,2356 |
|  |  | Subject | 524749064 | 10 | 52474906 | F (10, 10) = 0,5578 | P=0,8144 |
|  | **Holm-Šídák's multiple comparisons test** | **Mean Diff,** | **Below threshold?** | **Summary** | **Adjusted P Value** |  | |
|  | NaCl - c21 |  |  |  |  |  | |
|  | Baseline | 3728 | No | ns | 0,7068 |  |  |
|  | Test | 3736 | No | ns | 0,7068 |  |  |
|  | **Holm-Šídák's multiple comparisons test** | **Mean Diff,** | **Below threshold?** | **Summary** | **Adjusted P Value** |  |  |
|  | Baseline - Test |  |  |  |  |  |  |
|  | NaCl | 14230 | No | ns | 0,0576 |  |  |
|  | c21 | 14238 | No | ns | 0,0576 |  |  |
|  | VTA-ACC | **ANOVA table** | **SS** | **DF** | **MS** | **F (DFn, DFd)** | **P value** |
|  |  | Row Factor x Column Factor | 28020021 | 1 | 28020021 | F (1, 10) = 0,5035 | P=0,4942 |
|  |  | Row Factor | 53607987 | 1 | 53607987 | F (1, 10) = 0,9633 | P=0,3495 |
|  |  | Column Factor | 17259412 | 1 | 17259412 | F (1, 10) = 0,1572 | P=0,7001 |
|  |  | Subject | 1098170264 | 10 | 109817026 | F (10, 10) = 1,973 | P=0,1495 |
|  | VTA-OFC | **ANOVA table** | **SS** | **DF** | **MS** | **F (DFn, DFd)** | **P value** |
|  |  | Row Factor x Column Factor | 29953266 | 1 | 29953266 | F (1, 12) = 1,097 | P=0,3156 |
|  |  | Row Factor | 419113836 | 1 | 419113836 | F (1, 12) = 15,35 | P=0,0020 |
|  |  | Column Factor | 20150535 | 1 | 20150535 | F (1, 12) = 0,4847 | P=0,4996 |
|  |  | Subject | 498904844 | 12 | 41575404 | F (12, 12) = 1,523 | P=0,2387 |
|  | **Holm-Šídák's multiple comparisons test** | **Mean Diff,** | **Below threshold?** | **Summary** | **Adjusted P Value** |  | |
|  | Baseline - Test |  |  |  |  |  |  |
|  | NaCl | 9806 | Yes | ** | 0,0086 |  |  |
|  | c21 | 5669 | No | ns | 0,0652 |  |  |
|  | Baseline | 371,9 | No | ns | 0,9066 |  |  |
|  | Test | -3765 | No | ns | 0,425 |  |  |
| S4 B | Lever pressing | **ANOVA table** | **SS** | **DF** | **MS** | **F (DFn, DFd)** | **P value** |
|  |  | Time x Column Factor | 2904 | 1 | 2904 | F (1, 8) = 0,1174 | P=0,7407 |
|  |  | Time | 134316 | 1 | 134316 | F (1, 8) = 5,429 | P=0,0482 |
|  |  | Column Factor | 214866 | 1 | 214866 | F (1, 8) = 2,742 | P=0,1364 |
|  |  | Subject | 626989 | 8 | 78374 | F (8, 8) = 3,168 | P=0,0616 |
| S4 C | Lever pressing | **ANOVA table** | **SS** | **DF** | **MS** | **F (DFn, DFd)** | **P value** |
|  |  | Row Factor x Column Factor | 210170 | 5 | 42034 | F (5, 32) = 1,028 | P=0,4179 |
|  |  | Row Factor | 54897 | 1 | 54897 | F (1, 32) = 1,343 | P=0,2551 |
|  |  | Column Factor | 20702 | 5 | 4140 | F (5, 32) = 0,04629 | P=0,9986 |
|  |  | Subject | 2862085 | 32 | 89440 | F (32, 32) = 2,188 | P=0,0150 |
| S5 A | inputs | Unpaired t test |  |  | p=0,1542 | t=1,754, df=4 |  |
| S5 E | Initiation | ANOVA table | SS | DF | MS | F (DFn, DFd) | P value |
|  |  | Time x Column Factor | 1424 | 5 | 284,8 | F (5, 28) = 8,376 | P<0,0001 |
|  |  | Time | 1,519 | 1 | 1,519 | F (1, 28) = 0,04467 | P=0,8341 |
|  |  | Column Factor | 792 | 5 | 158,4 | F (5, 28) = 2,316 | P=0,0702 |
|  |  | Subject | 1915 | 28 | 68,39 | F (28, 28) = 2,012 | P=0,0348 |
|  | **Holm-Šídák's multiple comparisons test** | **Predicted (LS) mean diff,** | **Below threshold?** | **Summary** |  | | |
|  | Baseline |  |  |  |  |  |  |
|  | Ctrl: NaCl vs. Ctrl: c21 | 0 | No | ns |  |  |  |
|  | Ctrl: NaCl vs. ACC›CeA: NaCl | -2,333 | No | ns |  |  |  |
|  | Ctrl: NaCl vs. ACC›CeA: c21 | 3,5 | No | ns |  |  |  |
|  | Ctrl: NaCl vs. OFC›CeA: NaCl | -1,333 | No | ns |  |  |  |
|  | Ctrl: NaCl vs. OFC›CeA: c21 | -8,333 | No | ns |  |  |  |
|  | Ctrl: c21 vs. ACC›CeA: NaCl | -2,333 | No | ns |  |  |  |
|  | Ctrl: c21 vs. ACC›CeA: c21 | 3,5 | No | ns |  |  |  |
|  | Ctrl: c21 vs. OFC›CeA: NaCl | -1,333 | No | ns |  |  |  |
|  | Ctrl: c21 vs. OFC›CeA: c21 | -8,333 | No | ns |  |  |  |
|  | ACC›CeA: NaCl vs. ACC›CeA: c21 | 5,833 | No | ns |  |  |  |
|  | ACC›CeA: NaCl vs. OFC›CeA: NaCl | 1 | No | ns |  |  |  |
|  | ACC›CeA: NaCl vs. OFC›CeA: c21 | -6 | No | ns |  |  |  |
|  | ACC›CeA: c21 vs. OFC›CeA: NaCl | -4,833 | No | ns |  |  |  |
|  | ACC›CeA: c21 vs. OFC›CeA: c21 | -11,83 | No | ns |  |  |  |
|  | OFC›CeA: NaCl vs. OFC›CeA: c21 | -7 | No | ns |  |  |  |
|  | Test |  |  |  |  |  |  |
|  | Ctrl: NaCl vs. Ctrl: c21 | 0 | No | ns |  |  |  |
|  | Ctrl: NaCl vs. ACC›CeA: NaCl | -1,1 | No | ns |  |  |  |
|  | Ctrl: NaCl vs. ACC›CeA: c21 | 10,9 | No | ns |  |  |  |
|  | Ctrl: NaCl vs. OFC›CeA: NaCl | -3,433 | No | ns |  |  |  |
|  | Ctrl: NaCl vs. OFC›CeA: c21 | 15,73 | Yes | ** |  |  |  |
|  | Ctrl: c21 vs. ACC›CeA: NaCl | -1,1 | No | ns |  |  |  |
|  | Ctrl: c21 vs. ACC›CeA: c21 | 10,9 | No | ns |  |  |  |
|  | Ctrl: c21 vs. OFC›CeA: NaCl | -3,433 | No | ns |  |  |  |
|  | Ctrl: c21 vs. OFC›CeA: c21 | 15,73 | Yes | ** |  |  |  |
|  | ACC›CeA: NaCl vs. ACC›CeA: c21 | 12 | No | ns |  |  |  |
|  | ACC›CeA: NaCl vs. OFC›CeA: NaCl | -2,333 | No | ns |  |  |  |
|  | ACC›CeA: NaCl vs. OFC›CeA: c21 | 16,83 | Yes | ** |  |  |  |
|  | ACC›CeA: c21 vs. OFC›CeA: NaCl | -14,33 | Yes | * |  |  |  |
|  | ACC›CeA: c21 vs. OFC›CeA: c21 | 4,833 | No | ns |  |  |  |
|  | OFC›CeA: NaCl vs. OFC›CeA: c21 | 19,17 | Yes |  |  |  |  |
|  | **Holm-Šídák's multiple comparisons test** | **Predicted (LS) mean diff,** | **Below threshold?** | **Summary** | **Adjusted P Value** |  |  |
|  | Baseline - Test |  |  |  |  |  | |
|  | Ctrl: NaCl | -5,4 | No | ns | 0,4884 |  |  |
|  | Ctrl: c21 | -5,4 | No | ns | 0,4884 |  |  |
|  | ACC›CeA: NaCl | -4,167 | No | ns | 0,4884 |  |  |
|  | ACC›CeA: c21 | 2 | No | ns | 0,5572 |  |  |
|  | OFC›CeA: NaCl | -7,5 | No | ns | 0,1593 |  |  |
|  | OFC›CeA: c21 | 18,67 | Yes | **** | <0,0001 |  |  |
| S5 E | Maintenance | ANOVA table | SS | DF | MS | F (DFn, DFd) | P value |
|  |  | Time x Column Factor | 2715 | 5 | 543,1 | F (5, 28) = 3,374 | P=0,0165 |
|  |  | Time | 1545 | 1 | 1545 | F (1, 28) = 9,601 | P=0,0044 |
|  |  | Column Factor | 5642 | 5 | 1128 | F (5, 28) = 5,296 | P=0,0015 |
|  |  | Subject | 5966 | 28 | 213,1 | F (28, 28) = 1,324 | P=0,2314 |
|  | **Holm-Šídák's multiple comparisons test** | **Predicted (LS) mean diff,** | **Below threshold?** | **Summary** | **Adjusted P Value** |  |  |
|  | Baseline |  |  |  |  |  | |
|  | Ctrl: NaCl vs. Ctrl: c21 | -5,258 | No | ns | 0,9982 |  |  |
|  | Ctrl: NaCl vs. ACC›CeA: NaCl | 2,099 | No | ns | 0,9998 |  |  |
|  | Ctrl: NaCl vs. ACC›CeA: c21 | 2,581 | No | ns | 0,9998 |  |  |
|  | Ctrl: NaCl vs. OFC›CeA: NaCl | -6,421 | No | ns | 0,9947 |  |  |
|  | Ctrl: NaCl vs. OFC›CeA: c21 | 3,596 | No | ns | 0,9995 |  |  |
|  | Ctrl: c21 vs. ACC›CeA: NaCl | 7,357 | No | ns | 0,9913 |  |  |
|  | Ctrl: c21 vs. ACC›CeA: c21 | 7,84 | No | ns | 0,9909 |  |  |
|  | Ctrl: c21 vs. OFC›CeA: NaCl | -1,163 | No | ns | 0,9998 |  |  |
|  | Ctrl: c21 vs. OFC›CeA: c21 | 8,854 | No | ns | 0,9873 |  |  |
|  | ACC›CeA: NaCl vs. ACC›CeA: c21 | 0,4826 | No | ns | 0,9998 |  |  |
|  | ACC›CeA: NaCl vs. OFC›CeA: NaCl | -8,52 | No | ns | 0,9873 |  |  |
|  | ACC›CeA: NaCl vs. OFC›CeA: c21 | 1,497 | No | ns | 0,9998 |  |  |
|  | ACC›CeA: c21 vs. OFC›CeA: NaCl | -9,003 | No | ns | 0,985 |  |  |
|  | ACC›CeA: c21 vs. OFC›CeA: c21 | 1,015 | No | ns | 0,9998 |  |  |
|  | OFC›CeA: NaCl vs. OFC›CeA: c21 | 10,02 | No | ns | 0,9707 |  |  |
|  | Test |  |  |  |  |  |  |
|  | Ctrl: NaCl vs. Ctrl: c21 | -0,1846 | No | ns | 0,9831 |  |  |
|  | Ctrl: NaCl vs. ACC›CeA: NaCl | 4,846 | No | ns | 0,9462 |  |  |
|  | Ctrl: NaCl vs. ACC›CeA: c21 | 37,33 | Yes | *** | 0,0004 |  |  |
|  | Ctrl: NaCl vs. OFC›CeA: NaCl | -6,403 | No | ns | 0,9462 |  |  |
|  | Ctrl: NaCl vs. OFC›CeA: c21 | 19,69 | No | ns | 0,1807 |  |  |
|  | Ctrl: c21 vs. ACC›CeA: NaCl | 5,03 | No | ns | 0,9462 |  |  |
|  | Ctrl: c21 vs. ACC›CeA: c21 | 37,51 | Yes | *** | 0,0004 |  |  |
|  | Ctrl: c21 vs. OFC›CeA: NaCl | -6,218 | No | ns | 0,9462 |  |  |
|  | Ctrl: c21 vs. OFC›CeA: c21 | 19,88 | No | ns | 0,1807 |  |  |
|  | ACC›CeA: NaCl vs. ACC›CeA: c21 | 32,48 | Yes | ** | 0,0015 |  |  |
|  | ACC›CeA: NaCl vs. OFC›CeA: NaCl | -11,25 | No | ns | 0,6482 |  |  |
|  | ACC›CeA: NaCl vs. OFC›CeA: c21 | 14,85 | No | ns | 0,3766 |  |  |
|  | ACC›CeA: c21 vs. OFC›CeA: NaCl | -43,73 | Yes | **** | <0,0001 |  |  |
|  | ACC›CeA: c21 vs. OFC›CeA: c21 | -17,64 | No | ns | 0,2131 |  |  |
|  | OFC›CeA: NaCl vs. OFC›CeA: c21 | 26,09 | Yes | * |  |  |  |
|  | **Holm-Šídák's multiple comparisons test** | **Predicted (LS) mean diff,** | **Below threshold?** | **Summary** | **Adjusted P Value** |  |  |
|  | Baseline - Test |  |  |  |  |  | |
|  | Ctrl: NaCl | -0,2103 | No | ns | 0,9996 |  |  |
|  | Ctrl: c21 | 4,863 | No | ns | 0,9588 |  |  |
|  | ACC›CeA: NaCl | 2,537 | No | ns | 0,9807 |  |  |
|  | ACC›CeA: c21 | 34,54 | Yes | *** | 0,0004 |  |  |
|  | OFC›CeA: NaCl | -0,192 | No | ns | 0,9996 |  |  |
|  | OFC›CeA: c21 | 15,88 | No | ns | 0,1794 |  |  |
| S5 F | **Ctrl** | **ANOVA table** | **SS** | **DF** | **MS** | **F (DFn, DFd)** | **P value** |
|  |  | Row Factor x Column Factor | 11044963 | 1 | 11044963 | F (1, 8) = 0,6933 | P=0,4292 |
|  |  | Row Factor | 1040054 | 1 | 1040054 | F (1, 8) = 0,06528 | P=0,8048 |
|  |  | Column Factor | 1252772 | 1 | 1252772 | F (1, 8) = 0,04646 | P=0,8347 |
|  |  | Subject | 215708643 | 8 | 26963580 | F (8, 8) = 1,692 | P=0,2366 |
|  | **ACC-CeA** | **ANOVA table** | **SS** | **DF** | **MS** | **F (DFn, DFd)** | **P value** |
|  |  | Row Factor x Column Factor | 7456259 | 1 | 7456259 | F (1, 10) = 0,1650 | P=0,6932 |
|  |  | Row Factor | 719025 | 1 | 719025 | F (1, 10) = 0,01591 | P=0,9021 |
|  |  | Column Factor | 975247 | 1 | 975247 | F (1, 10) = 0,01467 | P=0,9060 |
|  |  | Subject | 664572537 | 10 | 66457254 | F (10, 10) = 1,470 | P=0,2767 |
|  | **OFC-CeA** | **ANOVA table** | **SS** | **DF** | **MS** | **F (DFn, DFd)** | **P value** |
|  |  | Row Factor x Column Factor | 8831723 | 1 | 8831723 | F (1, 10) = 0,3100 | P=0,5899 |
|  |  | Row Factor | 2810005 | 1 | 2810005 | F (1, 10) = 0,09865 | P=0,7599 |
|  |  | Column Factor | 3518141 | 1 | 3518141 | F (1, 10) = 0,1766 | P=0,6832 |
|  |  | Subject | 199243390 | 10 | 19924339 | F (10, 10) = 0,6995 | P=0,7088 |
| S5 G | Lever pressing | **ANOVA table** | **SS** | **DF** | **MS** | **F (DFn, DFd)** | **P value** |
|  |  | Time x Column Factor | 298407 | 5 | 59681 | F (5, 27) = 4,319 | P=0,0051 |
|  |  | Time | 147976 | 1 | 147976 | F (1, 27) = 10,71 | P=0,0029 |
|  |  | Column Factor | 570675 | 5 | 114135 | F (5, 27) = 1,078 | P=0,3946 |
|  |  | Subject | 2858626 | 27 | 105875 | F (27, 27) = 7,662 | P<0,0001 |
|  | **Holm-Šídák's multiple comparisons test** | **Predicted (LS) mean diff,** | **Below threshold?** | **Summary** | **Adjusted P Value** |  |  |
|  | B - T |  |  |  |  |  | |
|  | Ctrl: NaCl | -41,8 | No | ns | 0,6803 |  |  |
|  | Ctrl: c21 | 106 | No | ns | 0,5148 |  |  |
|  | ACC→CeA: NaCl | -77,17 | No | ns | 0,6038 |  |  |
|  | ACC→CeA: c21 | 249,5 | Yes | ** | 0,0052 |  |  |
|  | OFC→CeA: NaCl | 53,83 | No | ns | 0,6803 |  |  |
|  | OFC→CeA: c21 | 280,2 | Yes | ** | 0,0049 |  |  |
