# Supplementary material for: Optogenetic and chemogenetic approaches reveal differences in neuronal circuits that mediate initiation and maintenance of social interaction
Source: PLoS Biol. 2023 Nov 29;21(11):e3002343. doi: 10.1371/journal.pbio.3002343 (PMC10686636; doi:10.1371/journal.pbio.3002343)
Supplement: Attachment — Submitted filename: Rojek-Sito et al. Response to Reviewers_28.07.2023.pdf [file pbio.3002343.s010.pdf]

We express our gratitude to the reviewers for their valuable time and dedication in improving our manuscript, and we have provided our responses to their specific comments below, referencing the revised version of the manuscript

Reviewer #1

> *1. For the experiments inhibiting CeA-VTA in Figure 2D-G, the authors state that inhibition of the CeA-VTA disturbs the initiation and maintenance of social interaction. However, based on the figure, the change in interaction is only significantly different because the NaCl-receiving control partner rat show increased interaction. The number of approaches in the C21-treated CeA-VTA group does not seem different than the control groups, suggesting that blocking this circuit does not affect interaction but reduces maintenance and increases blocking. Could the authors elaborate on this discrepancy in the text? Could the increase in blocking be primarily caused by the increased interaction from the partner rat?*

Thank you for pointing out this issue. We found a difference in the initiation of social interaction between the focal rats, i.e., the ones with a blocked CeA-VTA pathway during the test, and their partners. However, we agree that the most visible changes in the behavior of focal rats were in maintenance and blocking, and they are statistically significant for both the comparison with the control group and the baseline. Further, as explained in detail below, we have decided to base our conclusions mostly on the comparison with the control group. Thus, we have changed the text accordingly, particularly highlighting a noticeable increase in the initiation of social contact by the partner rat resulting from the CeA-VTA projection inhibition in the focal rat:

‘We discovered that inhibiting the CeA-VTA pathway disrupts the maintenance of social interaction, specifically affecting positive reactions to the partner's approach, such as sniffing or allogrooming, and sustained close physical contact. Inhibiting this pathway leads to an increase in active blocking of social contact. At the same time, rats with inhibited CeA-VTA pathways initiate social interaction, approaching their partners as frequently as animals in the control group (Fig. 2D-G, S3D, E). Interestingly, the inhibition of the CeA-VTA pathway in one rat also alters the behavior of their partners, who attempt to initiate social contact more frequently than the control animals (Fig. 2D). Despite the increased initiation of social contacts by the partners, rats with inhibited CeA-VTA pathways do not respond to these attempts and actively avoid social interaction.’ (please see 167-176).

> *2. The authors observe that manipulating social circuits also affects the behavior of the partner rats. As all behaviors will at least partially depend on what the partner rat is doing, it may be useful to show the data as paired interactions instead of individual animals to more clearly show which of the animal is driving the change in behavior. However, this would not be essential to support the paper's conclusion.*

Thank you for your suggestion. We conducted an analysis of paired interactions; however, the data did not yield any additional insights into characterizing social interactions. As a result, we made the decision to present the behavior of the focal and partner animals separately.

Nevertheless, we acknowledge the significance of understanding the reciprocal influence between the interacting partners in order to comprehend the observed effects. Consequently, in the revised manuscript, we have expanded our discussion on these effects in greater detail. Please see the response to comment #1 regarding the CeA-VTA pathway and the excerpts from the text cited below. We consider the changes in the partners' behavior to be secondary to the changes observed in the treated rats.

‘We found that the activation of the VTA-ACC and VTA-OFC had a significant effect on the initiation of social contact but not on its maintenance (Fig. 3E- H, Fig. S3I). Stimulation of the VTA-ACC and VTA-OFC pathways increased active approaches toward the partners. However, simultaneously, rats with activated VTA-OFC projections more frequently blocked their partner's attempts to interact compared to control animals (Fig. 3G, H). These over-social and inconsistent behaviors during the activation of the VTA-ACC/OFC led to a decrease in positive responses to contact initiations and an increase in avoidance of social interaction in the partner rats (Fig. 3F-H, S3I).’ (please see 196-203).

‘Interestingly, this heightened sociability persisted despite the partners' attempts to avoid social contact, indicating potential challenges in recognizing and interpreting social signals from others in rats with overactive VTA-ACC/OFC pathways.’ (please see 317-319).

‘Specifically, we discovered that inhibition of the OFC-CeA pathway primarily impacts the initiation of social interaction. In contrast, inhibition of the ACC-CeA pathway predominantly affects the maintenance of social interaction when compared to the control group (Fig. 4H, I, K, S5E). Notably, when either the OFC-CeA or ACC-CeA pathways are inhibited, we observe an increase in attempts by the treated rats to block social interaction. This is evidenced by contact avoidance and pushing the partner away (Fig. 4J, K, Video 1, 2). The increased blocking behavior and decreased maintenance of social interaction in rats with an inhibited OFC-CeA pathway, compared to the partner rats, suggest additional challenges in maintaining social contact in the OFC-CeA group.’ (please see 228-237).

3. *Although the statistics in clearly stated in the figure, a statistics and data analysis section is missing from the method. A statistic section should be included to show if tests for normality were performed, if there were any exclusion criteria, and how significance was calculated.*

We apologize for this ommission in the previous version of the manuscript. In the corrected manuscript, we have addressed this issue by adding a dedicated statistics and data analysis section. Additionally, we have included a table presenting the results of all the statistical tests conducted (Table 2). In order to provide transparency, we now explicitly state the criteria for normality and exclusion in the respective section.

‘Data are presented as mean ± SEM or as the median and quartiles using boxplots. In cases where the data distribution deviated from normality (assessed using the Shapiro-Wilk test), non-parametric tests were employed. For comparing two groups, unpaired t-tests or Mann-Whitney tests were used, while paired t-tests were utilized for matched pairs comparisons. To compare more than two groups, ANOVA with repeated measures followed by Holm-Sidak post-hoc tests or Kruskall-Wallis tests followed by Dunn's tests (with FDR correction) were applied. Initiations, maintenance, and blocking during the test were assessed by comparing the values to the theoretical 0 using one-sample t-tests or Wilcoxon Signed Rank Test. The specific

statistical tests employed for each analysis are mentioned in the figure legends. We have included the most important findings in the text. Furthermore, Table 2 provides a comprehensive presentation of all the statistical comparisons that were conducted, including detailed results. GraphPad Prism version 9 (GraphPad Software, San Diego, CA) was used for all other statistical analyses. A significance level of $p < 0.05$ was considered statistically significant. The reasons for data exclusion were as follows: a) Misplaced injection/cannulation, which resulted in inaccurate targeting or delivery of substances. b) Loss of data due to tissue damage or malfunction of the data acquisition software.' (please see 728-743).

4. *Some of the investigated circuits appear more specific for social behavior, whereas others may more generally regulate reward as their manipulation also affects food reward. It would be helpful to more clearly state in the discussion which circuits are social-specific and some discussion on the distinction between more general versus specific circuit functions.*

Thank you for your suggestion. We have made the necessary updates to the Discussion section to explicitly state which circuits are specific to social interaction and differentiate between general circuits and those specific to social interaction:

'Our study provides evidence of the impact of manipulating the OFC-CeA, VTA-ACC, and VTA-OFC projections on the initiation of social contact. In contrast, manipulation of the ACC-CeA and CeA-VTA projections primarily affects responses to partners' attempts to initiate social interaction. This observation highlights the functional specificity of these circuits in the social domain.

To further investigate the circuits' involvement in social interaction, we conducted a comparative analysis with food motivation. Our aim was to determine whether these circuits play a general role in motivation or if their function is specific to social interaction. Surprisingly, our findings reveal that manipulating the CeA-VTA-ACC/OFC projections has no discernible impact on food motivation. However, it exerts a specific influence on social interaction. These results underline the specialized nature of these circuits in the social brain.' (please see 258-268).

Reviewer #2

1. *The author began to investigate the social cells in the CeA using Fos-activated virus expression. However, they did not study the projections of these social cells to the VTA. They used a retrograde Cav-cre and DIO-Gi strategy to label the projection neurons from the CeA to the VTA. We did not know how many of these cells were social cells. When studying the projection from the ACC or OFC to the CeA. They analyzed the ratio of the projection of ACC or OFC to the social cell in the CeA again. There is no direct functional evidence to show the circuits involved in social initiation and maintenance.*

Indeed, in this part of our study we examined the effects of manipulating the 'social' and 'food' CeA cells, however due to technical constraints, we did not track their projections in the optogenetic experiment (as the construct we used is primarily expressed in the cell bodies). Instead, we conducted an additional experiment showing that the CeA-VTA projection is

activated during social interaction. We utilized c-fos-Venus transgenic rats, injected with an anterograde transport tracer into the CeA, and subjected them to social interaction. Our findings revealed that a considerable number of the VTA neurons activated during social interaction receive projections from the CeA. This suggests that the pathway may play a causal role in social interaction. To test this hypothesis, we chemogenetically inhibited the projection. In the revised manuscript, we have now included the results of the projection tracking as we recognize that they lend support to our conclusions. Please refer to Fig S3A and the text: 'First, to investigate whether the CeA-VTA projection is activated by social interaction, we used c-fos-PSD95-Venus rats, which express a reporter protein under the control of the *c-fos* promoter, and injected them with an anterograde tracer (PHA-L: Phytohemagglutinin-L) into the CeA. Our findings revealed a dense projection from the CeA to the VTA cells activated during social interaction (Fig. S3A).' (please see 151-155).

Similarly, we tracked projections from the ACC and OFC onto the social and food cells, and then we tested the causal role of these projections chemogenetically.

The notable aspect of the c-Fos expression in the presented experiments is that it characterizes all neurons activated during social interaction, without specifically indicating their functional roles. Thus, the tracing tools we utilized did not enable us to differentiate between the neurons involved in the initiation and/or maintenance of social interaction. We acknowledge that this is a crucial issue that warrants further investigation in future studies. This would involve recording or live imaging of the activity of the CeA neurons with high temporal resolution.

2. The author claimed that the projection neurons from the CeA to the VTA are GABAergic. However, there is a lack of both morphological and electrophysiological evidence to support it.

3. The author used THcre mice (DA neurons) to investigate the function of the VTA-ACC and VTA-OFC pathways. Based on the results shown in Figure 2, the projection neuron of CeA sends its axon to VTA GABAergic neurons.

Thank you for bringing up these issues. Referring to comment #2. Our claim that the CeA projection to the VTA is GABA-ergic is supported by two key findings. Firstly, the activation of CeA social cells through photoactivation leads to a decrease in gamma-aminobutyric acid (GABA) levels in the VTA. Secondly, immunodetection of GABA neurons reveals that the majority of projections from the CeA ends at the GABA-ergic cells in the VTA. Together, these findings suggest inhibition of the GABA-ergic neurons in the VTA through GABA-ergic projections from the CeA. We acknowledge that this explanation may not have been adequately clarified in the previous version of the manuscript. Therefore, in the revised manuscript, we have included an additional explanation to ensure the clarity of our reasoning (quoted below).

Referring to comment #3. It appears that our line of reasoning was not clear in the previous version of the manuscript. Based on published research demonstrating that the GABA population within the VTA has a regulatory role in the activity of dopamine (DA) neurons within the VTA, we decided to manipulate the dopaminergic projection between the VTA and ACC/OFC. We have now provided a more detailed explanation of this rationale in the revised manuscript.

‘Next, we examined the colocalization of CeA inputs in the VTA with GAD67, a marker of GABAergic neurons. The analysis revealed a high degree of colocalization, indicating that the cells in the VTA receiving projections from the CeA are predominantly GABAergic (Fig. 2H, I). Further, activation of the CeA social cells led to a decrease of GABA in the VTA (Fig. S2). Together, these results suggest that the CeA output to the VTA may inhibit the tonic inhibition exerted by GABAergic interneurons on dopaminergic neurons. If this hypothesis holds true, the CeA-VTA projection should increase the activity of dopaminergic neurons in the VTA. In our next experiment, we investigated the role of dopaminergic projections from the VTA to the ACC and OFC in social interaction.’ (please see 181-189).

4. The author primarily utilized the DREADDs Gi strategy to verify the circuit's involvement in social interaction. It is better to confirm it using the DREADDs Gq strategy. For initiation and maintenance, real-time optogenetics may also be necessary.

We agree that our results can serve as a stimulus for future real-time optogenetic experiments, which would contribute to our understanding of the dynamics of the investigated circuits during social interaction. However, conducting such experiments would constitute a full new line of experiments focusing in detail on these aspects of behavior and would require introduction of imaging methodology not used so far in this study (not yet approved for this project by the Local Ethical Committee, a process that takes more time than that permitted for the revision of this manuscript).

5. The dopamine receptors are G-protein-coupled receptors, which means they do not directly modulate the neuronal activities of ACC and OFC neurons. The effect does rely on the type of dopamine receptor. What is the latency of VTA-ACC Gq experiments? It is better to provide evidence demonstrating that the neurons in the ACC and OFC are indeed activated by C21.

c21 had been administered i.p., 30 minutes before the experiment began, allowing sufficient time for the c21 to take effect and remain active throughout the entire interaction, as supported by the literature (Jendryka 2019) and our pilot studies (we have added the relevant explanation to the methods section: ‘The c21 was administered i.p., 30 minutes before the experiment began, allowing sufficient time for the c21 to take effect and remain active throughout the entire interaction, as supported by the literature (Jendryka 2019) and our pilot studies.’ (please see 540-543). While we acknowledge that gaining a deeper understanding of the receptors activated in the ACC and OFC would be highly interesting, this aspect will be addressed in future studies.

6. To determine whether the social and food cells overlap, the author could induce the expression of social and food cells at different stages using two different colors and count the number of cells.

Thank you for your comment. We attempted a method based on the *c-fos* expression and different colors; however, the results were not technically satisfying. Due to the use of rats in our studies, the options are limited in this regard. Therefore, unfortunately, we currently lack the necessary tools to conduct such experiments.

7. In Figure 2D, why do the No. approaches of the CeA-VTA:NaCl group and Control:NaCl group show a significant difference? It is the same as in Figure 3G.

The rats from the CeA-VTA:NaCl group (the partner rats injected with saline) engaged in interactions with rats whose CeA-VTA projection was inhibited. Their altered behavior likely reflects the changes in the behavior of the treated CeA-VTA:c21 rats, as the interaction is reciprocal and animals mutually influence each other. Similarly, in Fig 3G, attempts to avoid social interaction by the partners are shown. This behavior is likely caused by the changes in the focal rats' behavior, in which we manipulated the VTA-ACC or VTA-OFC projection activity. These rats exhibited heightened sociability, attempting to initiate social contact even when their partners tried to avoid it. We acknowledge that this aspect may not have been adequately explained in the previous version of the manuscript. Therefore, in the revised manuscript, we provide a more thorough discussion of these intriguing effects:

'Interestingly, the inhibition of the CeA-VTA pathway in one rat also alters the behavior of their partners, who attempt to initiate social contact more frequently than the control animals (Fig. 2D). Despite the increased initiation of social contacts by the partners, rats with inhibited CeA-VTA pathways do not respond to these attempts and actively avoid social interaction.' (please see 172-176).

'We found that the activation of the VTA-ACC and VTA-OFC had a significant effect on the initiation of social contact but not on its maintenance (Fig. 3E- H, Fig. S3I). Stimulation of the VTA-ACC and VTA-OFC pathways increased active approaches toward the partners. However, simultaneously, rats with activated VTA-OFC projections more frequently blocked their partner's attempts to interact compared to control animals (Fig. 3G, H). These over-social and inconsistent behaviors during the activation of the VTA-ACC/OFC led to a decrease in positive responses to contact initiations and an increase in avoidance of social interaction in the partner rats (Fig. 3F-H, S3I).' (please see 196-203).

'Specifically, we discovered that inhibition of the OFC-CeA pathway primarily impacts the initiation of social interaction. In contrast, inhibition of the ACC-CeA pathway predominantly affects the maintenance of social interaction when compared to the control group (Fig. 4H, I, K, S5E). Notably, when either the OFC-CeA or ACC-CeA pathways are inhibited, we observe an increase in attempts by the treated rats to block social interaction. This is evidenced by contact avoidance and pushing the partner away (Fig. 4J, K, Video 1, 2). The increased blocking behavior and decreased maintenance of social interaction in rats with an inhibited OFC-CeA pathway, compared to the partner rats, suggest additional challenges in maintaining social contact in the OFC-CeA group.' (please see 228-237).

8. The evidence of CeA predominantly projecting to VTA GABAergic neurons in Figure 2H-I is not strong enough.

Please see our answer to comment #3 of the review (above). We recognize that the clarity of the explanation may have been insufficient in the previous version of the manuscript. Consequently, in the revised manuscript, we have made a point to incorporate an additional explanation (please see 181-189) to ensure that our reasoning is presented with enhanced clarity.

9. *Do the same social cells in the CeA receive projections from both the ACC and OFC, or are they two distinct groups?*

The anterograde transport tracers used to track the ACC-CeA and OFC-CeA projections were injected into the ACC and OFC of separate animals (Fig. 4 B,C S5 D) , thereby preventing a direct comparison. However, our findings indicate that the ACC innervates a greater number of social cells in the CeA compared to the OFC. This suggests the possibility that certain cells may receive innervation from the ACC but not from the OFC. Nevertheless, a systematic comparison is required to provide a reliable answer to this question. We have included this explanation in the manuscript to acknowledge this. Please see lines 322-330 (' Inhibition of the OFC-CeA projection disrupted the initiation of social interaction, while inhibition of the ACC-CeA pathway disturbed its maintenance. The anterograde transport tracers used to track the ACC-CeA and OFC-CeA projections were injected into the ACC and OFC of separate animals, thereby preventing a direct comparison. However, our findings indicate that the ACC innervates a greater number of social cells in the CeA compared to the OFC. This suggests the possibility that certain cells may receive innervation from the ACC but not the OFC. However, a systematic comparison is necessary to gather evidence regarding whether the same CeA neurons receive projections from both the ACC and OFC.'

Minor issues:

10. *In Figure 1B, what is the total or average duration of social contacts?*

Thank you for bringing this lack of clarity to our attention. Fig. 1B displays the average number of social contacts. In contrast, the average duration of social contact is presented in Fig. S1 A. We have identified a minor error in the average contact time and have since corrected it. However, this correction does not alter the conclusion.

11. *Figure 1C lacks an image at low magnification to clearly show that the area is indeed CeA. Meanwhile, it is better to co-stain cFos with NeuN. What is the cell type of cFos-positive cells?*

We have included a lower magnification image in our manuscript (Fig. S1 B). Since c-Fos expression under physiological conditions is limited to neurons, we did not find it necessary to co-stain with NeuN. It is important to note that we did not specifically investigate the type of neurons in which c-Fos expression was observed.

12. *In Figure 3B, it appears that there are still some mCherry-positive signals that do not co-express with TH-positive cells. It is better to calculate the ratio of double positive cells to mCherry cells.*

Thank you for your suggestion. We have included the graph depicting the ratio of double-positive cells in our study (Fig. S3 G). The observed lack of complete co-expression is likely attributed to limitations in immunohistochemistry staining.

13. *In Figure 4G, there is a lack of low magnification images to show the infection area of ACC and OFC.*

Thank you for pointing out this omission. We have included images with lower magnifications in the corrected manuscript (Fig. S5 B). We specifically focused on the brain region displaying the expression of the DREADDs because, at lower magnifications, the expression is barely visible in print.

14. *Figure 3B shows that the TH+ cells are actually Cre-positive cells. It still needs to be stained with TH or DA to confirm that they are DA cells.*

We agree. In fact, Fig 3B displays the TH staining.

Reviewer #3

1. My main concern is on how the statistical analysis are performed, which might be affecting the conclusions that the authors obtain. ...the baseline was performed after a short isolation and test after 3 weeks of isolation. I do not think these conditions are comparable and the best way to question on the effects of their circuit manipulations. In my opinion, the most significant comparison would be paired samples t-tests between each animal of a pair on the test day. This will reveal which aspects of the social interaction are affected. Nevertheless, all the data provided from the control/baseline groups are very valuable and useful to see. With these graphs we understand that there are no previous differences between experimental groups, and can observe inter-individual variability, but I would not compare these values with the ones after 3w of social isolation. These data should be provided maybe in the supplementary materials, is useful to see, but omitted from the main figures, which might help also to simplify the main message.

We agree that deciding how to statistically analyze the data is not easy, and we are grateful to the reviewer for the suggestion. We have added the suggested analysis to what we have shown before and presented the results in the text and in the statistical table (Table 2), along with the results of other analyses.

In our study, we conducted three different types of comparisons: one with the control group, another with a partner, and the third with the baseline (after a short-time separation). We acknowledge that the comparison with the baseline may not be optimal due to the differing durations of social separation. However, comparing with a partner presents its own challenges as the partner's behavior is influenced by the behavior of the treated focal rats, creating interdependence between them. Consequently, we recognize that the comparison with the control group provides the most reliable insights.

However, we have also included the suggested comparisons with the partner rats as they provide valuable information. In response, we have thoroughly revised the manuscript, making necessary adjustments to the text and figures, in order to emphasize the primary conclusions

drawn from the comparison with the control group, as well as the conclusions derived from the changes in partners' behavior.

As suggested, we have also included the results of the third comparison for the sake of completeness. As rightly pointed out by the reviewer, the comparison with the baseline allows us to understand the changes in sociability that occurred during the experimental manipulations. However, we have moved these results to the supplementary material to focus the main conclusions on the comparison with the control group and the partners behavior.

Additionally, we now discuss attempts to block the interaction, as they may indeed represent the negative aspects of maintenance.

2. The other big concern I have, which evolves from the new way of doing statistics I suggest, is how the maintenance variable is currently calculated. As it is now, this variable is the sum of 2 variables: positive interactions ratio + crossings. My concern is specially on the calculation of the passive positive interactions normalizing by the number of initiations of the other animal. I understand the logic of this, but in this manner, in some cases, the effects seen in the maintenance are a consequence of the differences observed in the initiation of social interaction by the other animal. I would think that it would dissect better the contribution of each circuit if the variables (initiation vs maintenance) would not depend on each other. So I would propose to revisit the maintenance data with the number of passive interactions of the test only, not making it relative to the initiation variable, nor adding the crossings (as we can not disambiguate which animal of both perform the crossing and it adds the same amount equally to both animals of the pair). I would understand that a normalization to the frequency of initiation events of the partner would be performed if the duration of the passive approaches would be studied, but not so much in the frequency. Indeed, when the authors present the number of avoiding and pushing events, they are not normalizing by the number of initiations of the partner. I think that this new way of analyzing the data might provide cleaner differential effects of the maintenance variables (and circuits) which at the moment I fear are contaminated by how many initiations the partner does.

Thank you for bringing this issue to our attention. We have taken the following steps to address it. To accurately measure the motivation to maintain interaction, we have removed the number of crossings from the maintenance measure. We agree that crossings can reflect factors other than the motivation to maintain interaction. Instead, we have chosen to include only the positive responses to the partner's attempts to interact, as we believe they truly represent the motivation to maintain the interaction. However, we have retained the initiation by the partner factor, as we define maintenance as a positive response to the partner's attempt to interact. It is important to note that our initiation measure focuses on the initiation by the focal rat, while the maintenance measure specifically takes into account the initiation by the partner rat.

Therefore, the maintenance measure is now calculated as follows:

‘The positive reaction ratio (maintenance) was calculated by dividing the number of positive responses to the partner's approach by the number of the partner's approaches and multiplying by 100. A reaction was considered positive when the animal directed its head to the partner's body (nose to body, genital investigation), allowed for genital investigation or allogrooming, positively responded to pouncing (e.g., rolling onto its back or engaging in pinning), and/or wrestling (the on-top and on-bottom positions alternate during social play), or allowed the

partner to crawl over/under. Positive responses to the partner's approach did not include pushing or running away from the partner.' (please see 567-574)

We have included the corrected results in the revised manuscript for your review.

Minor comments

3. line 75: there is a missing "." just after the reference and Nevertheless

This sentence has been changed.

4. Fig 1B: where there any 22kH USVs in these first interactions after social isolation? If not, it would be interesting to comment this in the text, to support the statement that no agonistic interactions were observed, for example in line 95.

Thank you for this suggestion. No 22kHz USVs were observed. We have added this information to the revised manuscript: 'Consistently, the rats produced positive 50-kHz ultrasonic vocalizations (USVs), but they did not produce aversive 22-kHz USVs (Fig. 1A, B, S1A).' (please see 96-97).

5. LINE 109: just a style suggestion, as it is now, it is uncertain if "the previous work" refers to someone else's work of the first results that you have provided so far. Once you read the full sentence one can understand that it refers to previous literature. Maybe change the starting of this sentence to something like Previous work has shown.

We have changed the sentence to: 'Previous work has shown that optogenetic excitation of the CeA amplifies motivation to pursue food rewards in the progressive ratio lever pressing test [17].' (please see 112-113).

6. Fig S1: Figure legend (G) says the following "(G) Inhibition of both the food and social cells decreased lever pressing compared to baseline, in controls there was no difference between the baseline and following phases; two-way ANOVA (time effect: F(1.460,48,17) = 12.40, p=0.0002), followed by Holm-Sidak post-hoc tests." Does this F and p value refer to the all 4 groups? Does this mean that there is no interaction between the time and the experimental group? Because then it would mean that the decrease in the on-off period is also seen in the controls, meaning that the number of lever presses decreases over testing, which is expected. I understand that posthoc comparisons provide only significant differences in the "light" groups, however this posthoc comparisons are only justified if in the 2way ANOVA you have an interaction with time and experimental groups. In general, I find it confusing not to have all the statistical descriptives so we can see if the within or between subjects factors or the interactions are significant.. Could you please clarify me the rationale of only providing part of the statistical analysis? Maybe you are only providing the statistics that are significant to

simplify? I would be ok with that, but maybe it would be good to state it in the statistics section of the methods.

Thank you for pointing out this problem. Indeed, we only presented the most important comparisons in the text for clarity. To avoid confusion, we now have explicitly stated this in the statistics and data analysis section. Additionally, we have added a table (Table 2) that provides detailed results of all the statistical comparisons we conducted. Regarding the example you mentioned, the cited F and p values refer to the on/off periods.

7. Fig S2: at the end of the figure legend is written that Avg and sem is shown, however, in this figure, boxplots are used, which is indeed more informative, and correct in case of not normal distribution. Please correct, and indicate what the centroid line and whiskers indicate.

Thank you for drawing our attention to this mistake. We corrected it in the revised manuscript.

8. Regarding the Figures representing % [test-ctrl] in Initiation /Maintenance (for example fig 2G, fig3H, fig 4K). Why not to include here the Blocking results? (This comment will only hold if authors decide not to change the statistics as I suggested when articulating my main concerns). These are very helpful for providing a simplified view of the results. However, I wonder on whether the adequate statistics here would be to compare each group for each circuit against the 0 (meaning no change from baseline). As it is now, for example in fig3H the message is that VTA-ACC activation impacts more the initiation than the maintenance, and that difference is not significant in VTA-OFC. I wonder if the same message holds when asking if each circuit changes its own baseline (by differing from 0).

Thank you for this suggestion. We have made the following improvements based on your feedback. (1) We have added the Blocking results to all relevant graphs. As explained above, we are now comparing the results to the average results in the control groups. (2) We have included a comparison to the no-change level, treating it as additional information in analyzing raw data for initiation, maintenance, and blocking. These revisions have been incorporated into the manuscript.

9. While considering the initiation/maintenance/blocking results, I think is a good manner to explain different parts of social interaction, although they will be dependent on each other. For example, if one animal initiates many contacts, it might elicit more blockings by the partner. Did you explore correlations between these variables and how they increase or weaken for each circuit manipulations? For example, if inhibiting one circuit induces an animal to initiate less contacts, it might be that the partner will initiate more, and then the experimental animal will block more.

Thank you for this suggestion. We agree that this is an important point. In fact, we performed such an analysis. Unfortunately, the data are variable, and it appears that the groups are too

small to find statistically significant relationships between the number of attempts to initiate social contact by the partner and attempts to block them by the focal rat.

10. line 898: "Activation of both pathways results in active blocking of social contact by the partner". Could you please add at the end of this sentence "by the partner of the treated animals" or similar? I only realized that the slaine and the c21 animals were in the same pair after reading the full manuscript the first time and reaching the methods. It would help if this was stated at the beginning of the manuscript. Also, authors should think if they want to reduce the statistical comparisons to those that are relevant (here, for example, the activated circuit with c21 against their own control) instead of comparing everything with everything. If authors want to compare everything with everything, maybe corrections for multiple comparisons should be performed.

Thank you for pointing out this problem. We have added the phrase "by the partner of the treated animals" to clarify the source of the observed behavior. We have also provided a detailed explanation of the experimental design in the Results section. As explained previously, we have focused on comparing the results with the control groups and partners' behavior: ‘We performed three types of comparisons: with the control group, with a partner (who was treated with saline), and with the baseline (after a short-time separation). Our primary focus for drawing conclusions was the comparison with the control group and analyzing the changes in partners' behavior. Furthermore, we measured attempts to block the interaction as they represent the negative aspects of maintenance. The comparison with the baseline was conducted to monitor any potential alterations in sociability that may have arisen during the experimental manipulations. The results of these analyses can be found in the supplementary materials.’ (please see 158-166).

11. In general, please indicate in the figures whether the circuits are activated or inhibited, this would help readers to follow your results (for example fig s4).

We have implemented the relevant changes.

12. Fig S4: I am confused of the statistical comparisons used to obtain the conclusions regarding the social interactions. For example, in FifS4 D it is stated that: " Activation of VTA-ACC dopaminergic projection increases the initiation of social interaction. " However there are many more comparisons there that are statistically significant, also regarding the VTA-OFC. Could you please clarify? If the focus is on the within-subjects comparisons, then repeated measures anova should be used (baseline and test as within-subjects and treatment as between subjects). However, I am not sure if this is the best comparison to extract conclusions, as the values of a social interaction after 24h or after 3w isolation are going to differ. The baseline values are useful to see that prior manipulations the values of social interaction are similar, and to understand individual variability within each experimental group, but I would not take them as the statistical reference for the circuit manipulations.

Thank you for your suggestion. As mentioned earlier, we conducted three types of comparisons: one with the control group, another with a partner, and the third with the baseline (after a short-time separation). After careful consideration, we have decided to focus on the first two comparisons in the main text and figures. We have accordingly adjusted the text and figures to reflect this decision.

13. If within subjects (baseline/test) statistics are decided, then reorganize the graphs as in Fig S6B, where the 2 groups to compare are next to each other.

Thank you for this suggestion. We have now included the comparison to the baseline in the supplementary material. Please refer to Fig. S3 and Fig. S5.

14. Do the authors have USV data from the experiments of the circuits manipulations? It is only presented in Fig 1. If only USV data is available for the 1st figure, please specify this in the methods section.

We recorded USVs throughout all the experiments. However, most of the data does not show statistically significant differences, likely due to recording from two animals instead of individual subjects. We have added the relevant explanation to the methods section: 'The ultrasonic vocalizations (USVs) were recorded throughout all the experiments. However, it is important to note that the majority of the data did not exhibit statistically significant differences. We present only the statistically significant results of USVs analyses.' (please see 588-591).

15. In methods section, it is described that the behavioral data of Figure 1 was obtained studying interactions in the home-cage, and then, an open-field type cage was used, which improved tracking. Later, it is written that c-fos induced expression of channelrhodopsin was performed in a home cage, I imagine to reduce the expression of cfos while exposed to a novel environment. This is consistent for Figure 1, but where did the behavioral tests happened in Figure 4? Could you please clarify?

We apologize for the lack of clarity in the description of the methods. To address this, we have made revisions to the recording conditions description in the Methods section: 'Initially, the tests were performed in the home cage (the data presented in Fig. 1) and then in the interaction chamber (1m x 0.5 m), which improved the video recording quality (the data presented in Figs. 2, 3, 4, and S1).'(please see 465-467).

16. Line 485: it is stated that Bonsai was used to extract locomotor trajectories in social interaction tests. It would be nice that the authors explain here how did they created the workflow that allowed them to differentiate the trajectories of each individual animal (I assume using color), this is not trivial, and a short sentence describing how they performed this, would be useful. Moreover, did they use Bonsai to measure locomotor activity in the Food-progressive

ratio test? I see that time of exploration is presented in supplementary figures, but not sure how this was tracked.

Thank you for this suggestion. We added the relevant explanations to the methods section: ‘The animals were identified by observing the color markings on their backs. For each animal in each frame, the central position of the marked area was determined. To assess the activity of each animal, Euclidean distance measurement was utilized.’ (please see 579-582).

However, we did not perform this analysis for the progressive ratio test due to the technical limitations: ‘To measure cage exploration during the progressive ratio test, trained observers, who were blind to the experimental conditions, manually scored the data from behavioral experiments. BehaView software was utilized, providing frame-to-frame temporal resolution.’ (please see 634-636).

17. Line 497: Positive reaction ratio (maintenance). I was very confused the first time I read the manuscript, as only when I reached the methods definition I understood that this was a ratio. If at the end authors decide to include this ratio as their measure of maintenance of social interactions, it would be advisable that the write that is a ratio in the y axis legend of the figures. However, I thing that if the maintenance values are divided by the number of interactions, then they will be influenced by the INITIATION results.

Thank you for bringing this issue to our attention. We have made necessary changes to improve the accuracy of our calculations and modified the method of maintenance calculation. We have updated the description accordingly. Additionally, in the figures, we have clearly indicated that the values represented are ratios. Furthermore, we have included descriptions of the behavioral measures in the figures: ‘Initiation of social interaction: approaching a partner, Maintenance of social interaction: percentage of the positive reactions to partner’s approaches, Blocking of social interaction: pushing back or avoiding social interaction.’ (please see 1035-1038)

18. Line 561: I think there is an extra "." to be removed between cryostat (-20ºC) and Based on the rat brain atlas

19. Line 557: it is written that HPLC was performed in brains of animals after the progressive ratio test, however, they have performed them in animals after the social interaction test, too. Also, it is written that brains were cut into sections with a cryostat (please specify thickness), but not how the different brain areas were isolated for further neurochemical analyses.

Thank you for pointing out the need for a clearer description of the methods. To clarify, the HPLC analysis was exclusively conducted on the brains of animals that underwent the progressive ratio test. The social interaction, which led to c-fos expression, occurred one day prior to the HPLC analysis. The data pertaining to the manipulation of social cells was obtained from an experiment in which social cells (specifically, c-fos-tagged cells) were manipulated during a progressive ratio test. The thickness of the sections used in the analysis was 50 µm. We included these explanations in the methods section for comprehensive clarification: ‘The

data regarding the manipulation of social cells was obtained from an experiment in which specific social cells (c-fos-tagged cells) were manipulated during a progressive ratio test. Brain tissues were frozen in isopentane held on dry ice and stored at −76 °C for neurochemical analysis. Frozen brains were cut into 50 µm sections with a cryostat (−20 °C) based on the rat brain atlas of Paxinos and Watson (2007).' (please see 641-646).

20. Regarding these results, plotted in Figure S2, it would be advisable that the difference between ACC, OFC and PFC, as for my understanding both ACC and OFC could be also considered as prefrontal cortex. Is PFC referring to medial prefrontral cortex specifically (IL, Prelimbic).

Thank you for your suggestion. We have made the change from PFC to the medial prefrontal cortex (mPFC).

Reviewer #4

Major:

1a. The Introduction is rather poorly written and does not reflect the importance of the questions addressed in the study. In difference to many other studies that investigate social interactions in general, this study is focusing on functionally differentiating between two stages of social interaction - initiation and maintenance. Before addressing the putative involvement of the neural circuits, I would suggest to elaborate on the evolutionary importance of the two stages of social interaction.

We would like to thank the reviewer for providing valuable suggestions on improving the Introduction. As per the suggestion, we have included a paragraph that explains our motivation for investigating the neuronal circuits underlying the initiation and maintenance of social interaction:

'Here we aimed at dissecting whether neural circuits governing the motivation to initiate social contact and to maintain social interaction overlap or are distinct. The initiation of social contact necessitates the capacity to recognize and interpret social cues and signals from others, a motivation to engage in social interactions, and the willingness to physically approach others. On the other hand, the maintenance of social contact relies on the ability to synchronize behaviors, actions, and emotions with others, the reciprocation of social behaviors between individuals, and sustained engagement.

To explore this problem, we directed our attention to specific brain regions implicated in motivation and approach behaviors, such as the VTA and CeA, as well as regions associated with social cognition and executive functions, such as the ACC and OFC. We sought to investigate the causal role of this neural circuitry, characterized by its long-range functional connectivity, in different aspects of social interaction using a rat model. We analyzed multiple dimensions of social interaction, including initiation (reflecting the ability to initiate social contact), maintenance (reflecting the capacity to appropriately respond when a partner initiates social contact), and blocking (representing attempts to avoid social interaction initiated by the

partner). By examining these various aspects, we aimed to gain insights into the specific contributions of this circuitry to different facets of social behavior.' (please see 53-69).

We have also added a paragraph to further emphasize the significance of distinguishing between initiation and maintenance in the Discussion section:

'Understanding the specific circuits involved in initiating and maintaining social interaction can offer valuable insights into neurodevelopmental disorders such as autism spectrum disorder (ASD). The absence of social-seeking tendencies observed in individuals with ASD can be attributed to deficits in social motivation. Research has revealed that infants with a genetic predisposition to ASD exhibit diminished social motivation and interest. This deficiency fundamentally alters how individuals with ASD engage with and perceive the world, resulting in a lack of crucial opportunities to develop social perceptual and social cognitive abilities. Impaired social cognition and skills can thus be considered secondary effects stemming from reduced social motivation, potentially involving distinct neuronal circuits (Chevallier et al., 2012; Kohls et al., 2012; Zwaigenbaum et al., 2005). By addressing the core deficits in social motivation, there may be a possibility to enhance the development of social cognition and skills in individuals with ASD.' (please see 346-356).

We hope that with the changes made, the Introduction and Discussion now better reflect the significance of the questions addressed in our study.

1b. Furthermore, Line 81-83, the authors state: "Further, we explored the neuronal circuits underlying initiation, maintenance, and responses to partners' attempts to initiate contact, all of which have to be synchronized to build a successful social interaction" - in this study, no synchronization has been measured, please rephrase.

We apologize for our lack of precision. We have rephrased the sentence as follows:

'We analyzed multiple dimensions of social interaction, including initiation (reflecting the ability to initiate social contact), maintenance (reflecting the capacity to appropriately respond when a partner initiates social contact), and blocking (representing attempts to avoid social interaction initiated by the partner). By examining these various aspects, we aimed to gain insights into the specific contributions of this circuitry to different facets of social behavior.' (please see 64-69)

1c. Additionally, on lines 72-73 the authors mention that the CeA modulates emotion discrimination during social interaction but do not explain what that means and why it is relevant for the hypothesis. Please elaborate.

Thank you for pointing out the lack of clarity. We have added the following explanation:

'The VTA, OFC, and ACC have previously been associated with facilitating positive social interactions [3–9, 11]. The role of the CeA in driving social interactions remains largely unexplored. Previous studies have linked different subpopulations of neurons within the CeA to the modulation of rewarding experiences and the pursuit of food rewards [14,15] [16–19]. Furthermore, it has been also demonstrated that oxytocin signaling in the CeA modulates the

discrimination of emotions during social interactions [12,13], which is crucial for responding appropriately to the behavior of social interaction partners. The CeA exhibits strong connections with other structures in the mesocorticolimbic circuitry, and its stimulation recruits the VTA, NAcc, and OFC [14,20]. However, the functional distinction of CeA circuits based on their connectivity to different upstream and downstream brain areas remains unknown.' (please see 70-79).

2. The authors perform 3 weeks social separation prior to the test. While they are citing a paper (Gorlova et al, 2018) demonstrating that 3-weeks isolation does not lead to depressive-like disorders in rats, such a procedure certainly induces stress. Given that central amygdala is also involved in stress-related behavior and is affected by chronic stress, the authors will need to demonstrate that the increased CFos expression they observe is not related to the increased stress level. The experiment that should be conducted is following: social separation for 3 weeks after which the animals should be put in the context without social interaction - CFos expression level should be then compared with CFos expression during the social interaction. Also, the authors should cite the effects of stress throughout the text, in the introduction and discussion.

Thank you for your comment. According to the literature, exposure to any new environmental cues, including a new cage, induces some c-Fos expression in the brain, as c-Fos expression is triggered by any novel stimuli (Kaczmarek et al., 1988). Therefore, we believe that adding such experiments would not provide an answer to the question about the animals' stress levels.

However, we measured the general activity of the CeA after social separation, as well as the behavior of the animals, and we did not find any changes indicating an increased stress level. First, we assessed the basal level of c-Fos after three weeks of separation and did not observe elevated c-Fos expression in the CeA (please refer to Fig. 1F), in comparison to, for example, the not-separated home cage controls we used in other experiments. Second, the animals exhibited no signs of stress during interactions with other rats or when pressing the lever for food. Importantly, their behavior was comparable to that observed prior to the separation procedure (the baseline, measured after 24-hour separation).

3. While the results presented in Figure 1 related to social and food cells are interesting, I am somewhat confused regarding the authors' interpretation about the overlap between the two cell populations. As far understood, the activation is done in two groups, but then in inhibition experiments, the authors claim to inhibit both social and food cells simultaneously. How was that done? Please clarify. Did the authors conducted the social and food experiments in two different groups and looked into the Cfos expression in CeA? Does the expression pattern look similar in terms of C-Fos cell density and anatomical location? This might offer further insight into the potential overlap between the social and food cells, or lack thereof.

We apologize for the lack of clarity in the description of the experiments. We used c-fos-dependent genetic constructs to target opsins into behaviorally activated neurons that were activated either by social interaction or lever pressing for food. We divided the animals into four separate groups: food cells activation (expression of ChR stimulated by lever pressing for food); food cells inhibition (expression of NpHR stimulated by lever pressing for food); social

cells activation (expression of ChR stimulated by social interaction); and social cells inhibition (expression of NpHR stimulated by social interaction).

Next, the rats had the social or food cells activated or inhibited during lever pressing for food in the progressive ratio test, which was used to assess their food motivation. We observed similar effects of inhibiting the food and social cells (diminished food motivation), but different effects of activating the food and social cells (increased and diminished food motivation, respectively). Based on these findings, we conclude that at the functional level, the cells only partially overlap (if they were completely overlapping, we would expect the same effect of activation and inhibition for the social and food cells). This conclusion is further supported by the analysis of neurotransmitter release.

We have added additional explanations to the paragraph describing these experiments:

‘To further test the role of the CeA social cells and their behavioral specificity, we conducted another experiment to examine their involvement in motivation for food reward. Previous work has shown that optogenetic excitation of the CeA amplifies motivation to pursue food rewards in the progressive ratio lever pressing test [17]. Thus, we expected that if the social and food cells in the CeA overlap, their activation or inhibition will also affect food motivation. To test this, we induced the activity-dependent expression of opsins (specifically, the c-fos-ChR2 construct described above) in two groups of rats using the following methods: a) social interaction (social cells), and b) lever pressing for food (referred to as food cells, Fig. 1I-K, S1C). Then, we tested the effects of their photoactivation on motivation for food in the progressive ratio test. Consistently with the previous reports, we found that photoactivation of the food cells increased motivation for food, as measured by lever pressing. At the same time, activation of the social cells decreased it (Fig. 1K). As the photoactivation of the social cells promotes social interaction with the partner, this suggests that populations of social and food cells do not overlap completely.

Then, using the c-fos-NpHR construct, we targeted halorhodopsin into neurons that were activated either by social interaction or lever pressing for food. We repeated the experiment described above, this time photoinhibiting either the social cells or the food cells. We found that inhibiting both, food and social cells reduces motivation for the food reward and decreases cage exploration, suggesting some functional overlap between these populations (Fig. S1D-G). Notably, the behavior during sessions of social interaction or lever pressing for food used for induction of *c-fos*-dependent constructs did not differ between the groups (Fig. S1H-K). All the procedures were the same in the control groups, except that we did not use laser light during the test. The number of social and food cells activated in the CeA was comparable (Fig. S1J,K).’ (please see 124-133).

Regarding the anatomical location of the food and social cells, we found that they are not anatomically segregated within the CeA. We have added the relevant explanations to the text: ‘We did not observe anatomical segregation between the social and food cells.’ at lines please see 135-136. We hope that these additions clarify the experiment's design and the description of the results.

4. In the Methods section, line 541-545, the authors state: "To exclude the potential long-term effects of the optostimulation of the neuronal circuits in the CeA, we analyzed only the first two ON-OFF batches. We chose to do so because the subsequent laser activations introduced

significant fluctuations in lever pressing. For inhibition of the neurons in the CeA we quantified all 10 ON-OFF batches as the behavioral effects were stable over the training" The authors refer to their previous study where they show that the effect of activating opsin is less reliable. I found this explanation somewhat confusing and would like to see the data 3-10 ON-OFF batches for the activating condition.

Thank you for your comment. We have included a graph in the supplementary material (please refer to Fig. S1K) that represents the number of lever presses throughout the entire experiment for all groups.

5. *The experiments with measuring neurotransmitter release are interesting, but the reasoning behind this type of experiments as well as rationale to focus on specific pathway are insufficiently explained. Please elaborate on your rationale to focus on the decrease of GABA in VTA and not, for example on the decrease of Glu or NA in PFC. Also, the authors did not perform any measurements in Nucleus Accumbens, which also receives CeA inputs, please clarify.*

Thank you for pointing out this lack of clarity. We have indeed focused on specific results from the analysis of neurotransmitter release. Specifically, we chose to emphasize the CeA-VTA pathway and the release of GABA in the VTA. This emphasis stemmed from the tracing experiment we conducted, which demonstrated high activity in this projection during social interactions. The changes observed in the VTA led us to speculate that the dopaminergic projection could be involved, and interestingly, we also observed changes in dopamine levels in the ACC. However, we acknowledge that the mPFC and NAc are likely important players in the neuronal circuits controlling social behavior, and they warrant further studies. In response to your feedback, we have included these explanations in the revised manuscript:

‘Comparing the levels of neurotransmitters in different brain structures after the stimulation of food and social cells, we observed changes in neurotransmitter release in the VTA, ACC, OFC, PFC, and NAc following the activation of social cells but not food cells (Fig. S2). We directed our focus towards the CeA-VTA-ACC/OFC pathways. This emphasis stemmed from the tracing experiment we conducted, which demonstrated high activity of the CeA-VTA projection during social interactions (see below). Furthermore, the changes observed in the VTA led us to speculate that the dopaminergic projection could be involved. Accordingly, we observed changes in dopamine levels in the ACC.’ (please see 141-148)

‘First, to investigate whether the CeA-VTA projection is activated by social interaction, we used c-fos-PSD95-Venus rats, which express a reporter protein under the control of the *c-fos* promoter, and injected them with an anterograde tracer (PHA-L: Phytohemagglutinin-L) into the CeA. Our findings revealed a dense projection from the CeA to the VTA cells activated during social interaction (Fig. S3A).’ (please see 151-155)

Additionally, we have incorporated the data on the release of neurotransmitters in the NAcc in the current version of the manuscript. Please refer to Figure S2 for the corresponding results.

6. Dopaminergic VTA-ACC - the initiation effect for VTA-ACC is similar to the maintenance effect of VTA-OFC (Figure 3 F). This was not mentioned/discussed. Further, in H - in the legend inhibition is mentioned although the figure appears to contain only activation - please clarify. In addition, no reference to this result (graph H) is given in the text.

Thank you for pointing out this issue. In our study, we conducted three different types of comparisons: one with the control group, another with a partner, and the third with the baseline (after a short-time separation). We acknowledge, as suggested by another reviewer, that the first comparison, specifically the one with the control group, provides the most valuable insights. Therefore, we have revised the manuscript and adjusted the text and figures to emphasize the main conclusions derived from this particular comparison. We have discussed all these results in the revised manuscript. We apologize for the mistake in the legend describing the results shown in Fig. 3H and the lack of reference in the text. We have corrected these errors in the current version of the manuscript.

Minor

7. Highlight 2: please clarify

We re-wrote the highlight. Now it is as follows: 'Social behavior-mediating sub-circuits are defined by their connectivity'. (please see 25)

8. Line 107: "...CeA cells causally involved...", so far the experiment does not delve into activating or inhibiting these cells, so the authors should use "associated" instead.

This sentence actually refers to the optogenetic experiment in which we activated the CeA social cells and as a result observed an increase in social contacts.

9. Lines 130-131: Sentence is confusing, please clarify.

We rewrote the sentence. Now it is: 'We did not observe anatomical segregation between the social and food cells. Therefore, to investigate the factors that differentiate the social and food cells, we focused on identifying the CeA outputs of these cell populations.' (please see 135-137)

10. Lines 134-136: The rationale behind the next experiments are explained here, but in a rushed way. Please elaborate.

We re-wrote the paragraph. Now it is as follows. 'Comparing the levels of neurotransmitters in different brain structures after the stimulation of food and social cells, we observed changes in neurotransmitter release in the VTA, ACC, OFC, PFC, and NAc following the activation of social cells but not food cells (Fig. S2). We directed our focus towards the CeA-VTA-ACC/OFC pathways. This emphasis stemmed from the tracing experiment we conducted, which demonstrated high activity of the CeA-VTA projection during social interactions (see below). Furthermore, the changes observed in the VTA led us to speculate that the

dopaminergic projection could be involved. Accordingly, we observed changes in dopamine levels in the ACC.' (please see 141-148).

11. Lines 154-155: Please elaborate how (with double labeling?)

We explained. Now it is: 'Next, we examined the colocalization of CeA inputs with GAD67, a marker of GABAergic neurons. The analysis revealed a high degree of colocalization, indicating that the cells in the VTA receiving projections from the CeA are predominantly GABAergic neurons'. (please see 181-184).

12 Lines 156-158: The authors want here to suggest that the CeA output to VTA inhibit the tonic inhibition of dopaminergic neurons via gabaergic interneurons, but the text is confusing. It should be clarified.

We have rewritten this paragraph. The revised version reads as follows: 'The VTA, a key source of dopamine in the cortex, plays a crucial role in encoding motivation for social interactions [30,31]. It is a heterogeneous region composed of various cell types, including neurons that release dopamine (DA), GABA, or glutamate [31,32]. The activity of DA neurons is regulated by GABAergic interneurons [31]. Our findings demonstrate that the CeA-VTA projection, which primarily targets GABAergic neurons in the VTA, mediates the maintenance of social interaction, likely through disinhibition of DA neurons in the VTA. However, further studies are necessary to elucidate the interactions between CeA projecting neurons and VTA GABAergic and DA neurons that drive social behavior.' (please see 301-308).

13. Lines 212-214: Sentence is confusing and poorly written. Please correct.

We have rewritten the entire paragraph, including the indicated sentence. The revised version is now: 'Successful social interaction relies on two fundamental conditions. Firstly, individuals must display the motivation to initiate interaction and an ability to recognize and interpret social cues and signals from others in order to initiate social contact. Secondly, they need the skills and capability to sustain the interaction for a time sufficient to synchronize behaviors, actions, and emotions with others. Consequently, when analyzing the behavioral outcomes of the manipulation of such circuit activity, we comprehensively examined various aspects of social interaction, encompassing both its initiation and maintenance. By doing so, we aimed to gain insights into the distinct neural circuits that underlie the diverse skills required for initiating and maintaining social interaction. Furthermore, we classified avoidance of social interaction and pushing back the partner rat as a third category of social interaction, referred to as "blocking." This category represents the unwillingness or aversion to engage in social contact and can be considered an attempt to prevent both the initiation of social interaction by others and its maintenance.' (please see 245-257).

14. Line 229: …whole role…should be: which role

We have rewritten the sentence. The revised version is now: 'The CeA is part of the reward system, and its role in processing food and drug rewards is well-established [14]' (please see 277-278).

15. Lines 252-254: Please elaborate here and cite the studies.

We rewrote the sentence and cited the relevant studies. Now it reads as follows: 'Previous studies have demonstrated that the processing of social stimuli involves dopamine receptors [33] in the ACC and OFC [34]. In particular, recent research suggests that the dopamine system in the OFC may play a crucial role in the development of social anxiety disorder [34]. Furthermore, studies have indicated that the participation of dopamine D2 receptors in the ACC is essential for processing social information during social observational fear learning in animals [33].' (please see 309-314).

16. Lines 259-261: Sentence is confusing, please clarify.

We have rewritten the sentence. The revised version reads as follows: 'Our results provide evidence that distinct neuronal circuitry mediates the different abilities required for initiating and maintaining social contact. Additionally, we demonstrate that manipulating neuronal projections involved in the initiation and/or maintenance of social interaction can impact not only the behavior of the treated animals but also the response of their interaction partners. This can manifest as the discontinuation of interaction and aversion to social contact, potentially indicating abnormalities in the behavior of the treated rats.' (please see 332-338).
